# The Asian arowana (*Scleropages formosus*) genome provides new insights into the evolution of an early lineage of teleosts

**DOI:** 10.1038/srep24501

**Published:** 2016-04-19

**Authors:** Chao Bian, Yinchang Hu, Vydianathan Ravi, Inna S. Kuznetsova, Xueyan Shen, Xidong Mu, Ying Sun, Xinxin You, Jia Li, Xiaofeng Li, Ying Qiu, Boon-Hui Tay, Natascha May Thevasagayam, Aleksey S. Komissarov, Vladimir Trifonov, Marsel Kabilov, Alexey Tupikin, Jianren Luo, Yi Liu, Hongmei Song, Chao Liu, Xuejie Wang, Dangen Gu, Yexin Yang, Wujiao Li, Gianluca Polgar, Guangyi Fan, Peng Zeng, He Zhang, Zijun Xiong, Zhujing Tang, Chao Peng, Zhiqiang Ruan, Hui Yu, Jieming Chen, Mingjun Fan, Yu Huang, Min Wang, Xiaomeng Zhao, Guojun Hu, Huanming Yang, Jian Wang, Jun Wang, Xun Xu, Linsheng Song, Gangchun Xu, Pao Xu, Junmin Xu, Stephen J. O’Brien, László Orbán, Byrappa Venkatesh, Qiong Shi

**Affiliations:** 1 Shenzhen Key Lab of Marine Genomics, Guangdong Provincial Key Lab of Molecular Breeding in Marine Economic Animals, Shenzhen 518083, China; 2 BGI-Shenzhen, Shenzhen 518083, China; 3 Key Laboratory of Tropical & Subtropical Fishery Resource Application & Cultivation, Ministry of Agriculture, Pearl River Fisheries Research Institute, Chinese Academy of Fishery Sciences, Guangzhou 510380, China; 4 Institute of Molecular and Cell Biology, A^*^STAR, Biopolis, Singapore 138673, Singapore; 5 Reproductive Genomics Group, Temasek Life Sciences Laboratory, Singapore 117604, Singapore; 6 Laboratory of Chromosome Structure and Function, Department of Cytology and Histology, Biological Faculty, Saint Petersburg State University, Saint-Petersburg 198504, Russia; 7 Realbio Genomics Institute, Shanghai 200050, China; 8 Theodosius Dobzhansky Center for Genome Bioinformatics, Saint Petersburg State University, St. Petersburg 199004, Russia; 9 Institute of Molecular and Cellular Biology, Siberian Branch of the Russian Academy of Sciences, Novosibirsk 630090, Russia; 10 Novosibirsk State University, Novosibirsk 630090, Russia; 11 Genomics Core Facility, Institute of Chemical Biology and Fundamental Medicine, Siberian Branch of the Russian Academy of Sciences, Novosibirsk 630090, Russia; 12 Environmental and Life Sciences Programme, Faculty of Science, Universiti Brunei Darussalam, BE1410 Brunei Darussalam; 13 James D. Watson Institute of Genome Science, Hangzhou 310008, China; 14 Princess Al Jawhara Center of Excellence in the Research of Hereditary Disorders, King Abdulaziz University, Jeddah, Saudi Arabia; 15 Department of Biology, University of Copenhagen, DK-2200 Copenhagen, Denmark; 16 Dalian Ocean University, Dalian 116023, China; 17 Freshwater Fisheries Research Center, Chinese Academy of Fishery Sciences, Wuxi 214081, China; 18 BGI-Zhenjiang Institute of Hydrobiology, Zhenjiang 212000, China; 19 Oceanographic Center, Nova Southeastern University Ft. Lauderdale, Ft Lauderdale, Florida 33004, USA; 20 Department of Animal Sciences and Breeding, Georgikon Faculty, University of Pannonia, H-8230 Keszthely, Hungary; 21 Centre for Comparative Genomics, Murdoch University, Murdoch, 6150 Australia

## Abstract

The Asian arowana (*Scleropages formosus*), one of the world’s most expensive cultivated ornamental fishes, is an endangered species. It represents an ancient lineage of teleosts: the Osteoglossomorpha. Here, we provide a high-quality chromosome-level reference genome of a female golden-variety arowana using a combination of deep shotgun sequencing and high-resolution linkage mapping. In addition, we have also generated two draft genome assemblies for the red and green varieties. Phylogenomic analysis supports a sister group relationship between Osteoglossomorpha (bonytongues) and Elopomorpha (eels and relatives), with the two clades together forming a sister group of Clupeocephala which includes all the remaining teleosts. The arowana genome retains the full complement of eight Hox clusters unlike the African butterfly fish (*Pantodon buchholzi*), another bonytongue fish, which possess only five Hox clusters. Differential gene expression among three varieties provides insights into the genetic basis of colour variation. A potential heterogametic sex chromosome is identified in the female arowana karyotype, suggesting that the sex is determined by a ZW/ZZ sex chromosomal system. The high-quality reference genome of the golden arowana and the draft assemblies of the red and green varieties are valuable resources for understanding the biology, adaptation and behaviour of Asian arowanas.

With over 30,000 extant species, ray-finned fishes (Class: Actinopterygii) are the largest group of extant vertebrates. The species-richness of this group is in fact largely due to a single monophyletic group of fishes, the teleosts, which account for more than 99% of present day ray-finned fishes^1^. Teleosts are also the most diverse group of vertebrates that exhibit a wide diversity in their morphology, colouration, behaviour and adaptations. The evolution of this group of ray-finned fishes was accompanied by emergence of several novel characters which were crucial to their success^2^. These include replacement of bony plates and denticles with overlapping disc-like scales which reduced the weight and increased flexibility, the use of a swim bladder to maintain buoyancy, replacement of heavy toothed jaws with more flexible and protractile ones for efficient prey capture, shift from a heterocercal to homocercal tail for faster speed and change in the position of the paired fins for better maneuverability^2^. Sequencing and analysis of whole-genomes from fugu and other teleosts have revealed that the common ancestor of teleosts experienced a whole-genome duplication event, known as the teleost-specific genome duplication (TGD), about 350 million years ago^3^,^4^. The TGD has been proposed to have provided the additional raw genetic material that was used for evolving genes with novel functions resulting in novel phenotypes thereby spurring the diversity of teleosts^5^,^6^. Thus, genome sequences of ancient groups of teleosts that emerged soon after the TGD are crucial for understanding the genetic basis of the origin and diversity of teleosts. Teleosts are classified into three broad groups: the Osteoglossomorpha (bonytongues and mooneyes), Elopomorpha (eels and relatives) and Clupeocephala (the remaining teleosts). Among them, Osteoglossomorpha is considered as one of the ancient groups, with fossil records dating back to the late Jurassic^7^. However, the phylogenetic relationships of the three groups are controversial^8^,^9^,^10^,^11^,^12^,^13^. Earlier mitogenome-based phylogenetic analysis had placed Osteoglossomorpha as the ancestral teleost group, with Elopomorpha and Clupeocephala forming a monophyletic group^11^. However, recent studies based on nuclear genes^8^,^13^ and ultraconserved elements^10^ have suggested Elopomorpha as the most ancestral teleost group. Recently, a study based on phylogenetic analysis of ‘question-specific’ genes showed a sister relationship of Osteoglossomorpha with Elopomorpha^9^.

The Asian arowana or dragonfish (Scleropages formosus; Order Osteoglossiformes; hereafter referred to as arowana) is a member of the superorder Osteoglossomorpha and also one of the most expensive cultured ornamental fishes. Currently it is listed under the Convention on International Trade in Endangered Species of Wild Fauna and Flora (CITES) Appendix I, the only commercially cultured species included in the list^14^. Three major colour varieties of arowana occur naturally: the green, golden and red varieties. However, the molecular mechanisms underlying these colour variations remain largely elusive. In order to reduce the pressure on natural populations, farms have started culturing different varieties of arowana. One of the bottlenecks in the cultivation of arowana is the lack of a reliable and easy sex-determining method. Since the sex of even mature brooders is not obvious, broodstock selection and management often become skewed towards selection by desired phenotype, like colour and body shape. Thus, there is a need to understand the sex determination mechanism in arowana and develop a viable sexing method that can be used at an early developmental stage of the fish.

In this study, we have generated a high quality, chromosome-level reference genome of a golden female arowana (Fig. 1A), in addition to draft genome sequences of red and green arowana varieties (Fig. 1B,C). Based on a stringent, genome-scale set of one-to-one orthologues from various teleosts, we report a robust phylogenomic analysis which resolves the branching order of the three major lineages of teleosts. Our study also identifies differentially expressed genes potentially involved in the colour variations, and provides the first indication for the likely presence of a ZW/ZZ sex chromosomal system in the species.

## Results and Discussion

### Reference genome assembly and annotation

**Figure 2:**
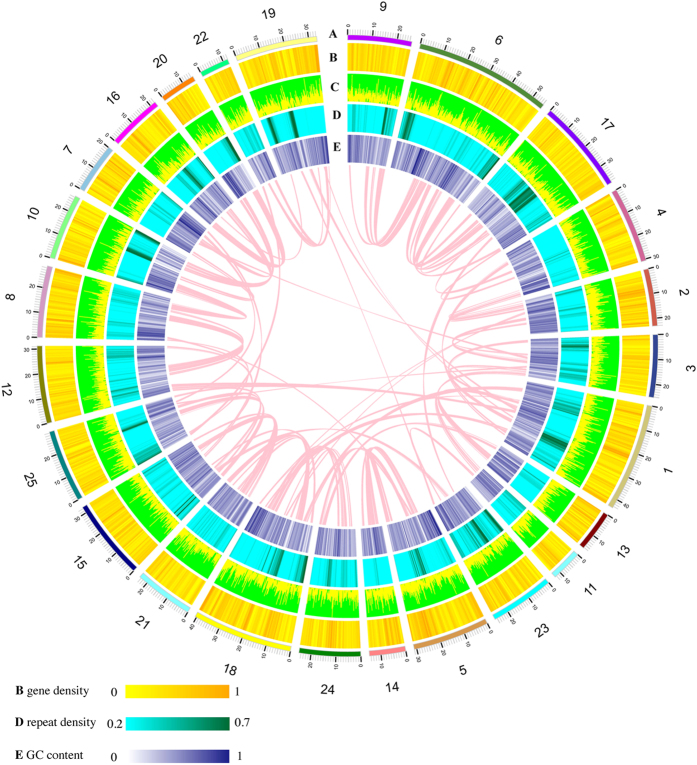
Characteristics of the reference genome of the golden Asian arowana. Concentric circles from the outside: (**A**) Chromosome length (Mb) and numbers. Chromosome numbers were assigned based on the linkage groups. (**B**) Distribution of gene density in 1Mb non-overlapping windows. (**C**) Expression level of genes in skin tissue of the golden arowana. High yellow peaks indicate strong expression. (**D**) Distribution of repeat density in 1Mb non-overlapping windows. Deeper green colour indicates higher repeat density. (**E**) Distribution of GC content in 1Mb non-overlapping windows. Darker blue colour indicates higher GC content. The pink lines represent the inner synteny blocks.

Recently, a draft assembly of an Asian arowana (colour variety unknown) with an N50 scaffold length of 59 kb has been generated^15^. We sequenced the genomes of golden, red and green varieties of arowana to >100-fold coverage (Supplementary Tables 1 and 2) using the Illumina HiSeq2000 platform. Sequence reads from the three varieties were assembled separately (Supplementary Table 3) using SOAPdenovo2^16^ resulting in N50 scaffold sizes of 5.96, 1.63 and 1.85 million bases (Mb), and genome assemblies spanning approximately 779, 753 and 759 Mb for the golden, red and green varieties, respectively, in agreement with their estimated genome sizes based on k-mer analyses (Table 1 and Supplementary Fig. 1). Evaluation using CEGMA^17^ and de novo-assembled transcripts showed that the three assemblies covered over 98% of core eukaryotic genes and 95% of gene regions (Supplementary Tables 4 and 5), confirming their high level of completeness and accuracy. We then indentified 829,293, 1,168,314 and 1,684,422 heterozygous single nucleotide polymorphisms (SNP) and detected the following heterozygosity levels: 1.01%, 1.23% and 1.88% in the golden, red and green arowana genomes, respectively. To further improve the quality of the golden variety genome assembly, we developed a high-density genetic map by restriction site-associated DNA sequencing (RAD-seq) using 94 F2 individuals that originated from red grade 1 and Malaysian golden arowana grandparents^18^. Subsequently, we identified 22,881 SNPs using the golden assembly as the reference, of which 5,617 refined SNPs and their corresponding scaffolds were clustered and embedded into 25 linkage groups (Fig. 2) spanning approximately 3,240 cM and 683 Mb (87.7% of the golden variety assembly; Supplementary Fig. 2 and Supplementary Table 6). The high-quality linkage group-anchored assembly of the golden variety of Asian arowana can be used as a reference genome, whereas the draft genomes of green and red varieties are suitable for comparative studies.

Transposable elements (TEs) account for 27–28% of the three genomes (Supplementary Table 7), with the TcMar DNA transposon being the most predominant type (approximately 8%). We predicted 22,016, 21,256 and 21,524 genes in the genome assemblies of golden, red and green varieties, respectively (Table 1), using a combination of *de novo*, homology- and transcriptome- (comprising 4 Gb transcriptome reads from the skin of each colour variety) based annotation methods (Supplementary Table 8). Approximately 95% of genes from each variety had both transcriptome and homology support (Supplementary Fig. 3), and about 87% of the genes showed evidence for expression (FPKM >0) (Supplementary Table 9). Gene structures of the three genomes were in high accordance with those of other fishes (Supplementary Fig. 4), and over 96% of them possessed at least one function that can be assigned based on sequences in public databases (Swiss-Prot, TrEMBL^19^, Gene Ontology^20^ and KEGG^21^, Supplementary Table 10). All of the above data confirmed the accuracy and high quality of the three gene sets. The distribution of gene and repeat density, GC content, and gene expression levels in skin tissue across the chromosomes of golden variety are shown in Fig. 2.

### Branching order of Osteoglossomorpha, Elopomorpha and Clupeocephala

**Figure 3:**
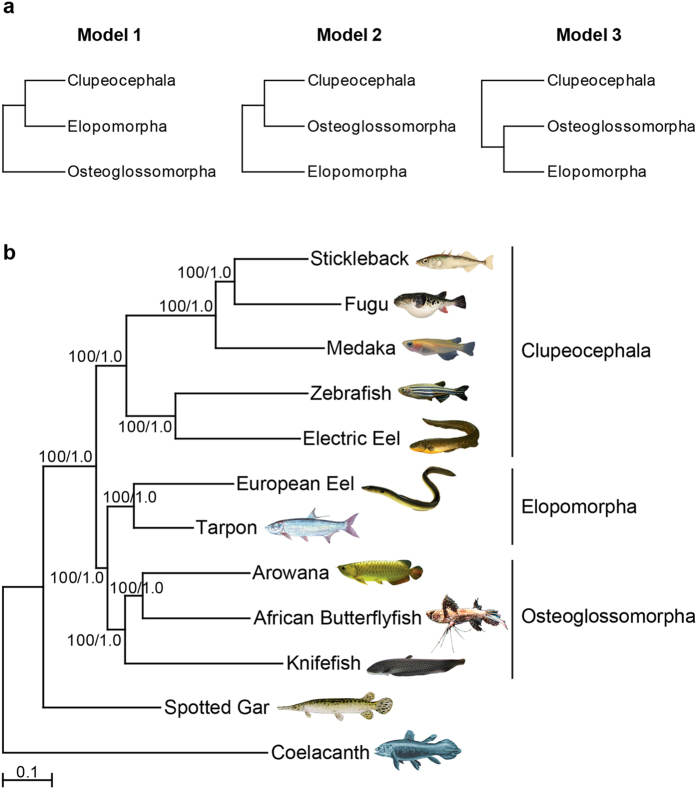
Phylogenetic relationship of the Asian arowana to other teleosts. (**A**) Alternative phylogenetic models for the branching order of Osteoglossomorpha, Elopomorpha and Clupeocephala. (**B**) Phylogenetic position of Asian arowana with respect to other teleost fishes. The trees are based on 418 one-to-one orthologues (294,783 nucleotide positions) from 12 vertebrates. Values shown at the nodes are Maximum Likelihood bootstrap percentages/Bayesian posterior probability values. The scale bar represents 0.1 substitutions per site.

Previous studies based on morphological and molecular data have produced conflicting phylogenetic relationships among the three major lineages of teleosts. Morphological and fossil data have placed osteoglossomorphs^22^ or elopomorphs^23^ as the sister group to the remaining teleosts. Phylogenetic analysis based on 28S ribosomal RNA as well as a larger set of 4,682 protein-coding genes from jawed vertebrates placed elopomorphs as sister to osteoglossomorphs^9^,^12^. On the other hand, mitogenome-based phylogenetic analysis^11^ as well as a study based on 170 proteins extracted from a draft assembly of the Asian arowana^15^ have placed osteoglossomorphs as sister to the remaining teleosts. More recent phylogenetic studies based on nuclear genes^8^,^13^ or ultraconserved elements^10^ from a wide range of teleosts have suggested that Elopomorpha is the sister group to the remaining teleosts (Fig. 3A).

The availability of a high quality reference genome of the arowana provided us the opportunity to analyze the branching order of the three groups using a phylogenomic approach. We generated Maximum Likelihood (ML) and Bayesian Inference (BI) trees using 2,463 one-to-one orthologues from nine species including one elopomorph, one osteoglossomorph, five clupeocephalans and two outgroups (spotted gar and coelacanth). Both ML and BI methods gave identical topologies with strong ML bootstrap (BS) and Bayesian posterior probability (PP) support values (Supplementary Fig. 5). Both trees showed an “Elopomorpha sister to Osteoglossomorpha” topology with maximal support (BS 100, PP 1.0) (Supplementary Fig. 5). The monophyletic clade comprising elopomorphs and osteoglossomorphs formed a sister group to Clupeocephala, again with maximal support (BS 100, PP 1.0; Supplementary Fig. 5). To evaluate the likelihoods of alternate tree topologies, we performed topology testing using CONSEL (Supplementary Table 11). Indeed, the ‘Elopomorpha sister to Osteglossomorpha’ arrangement appeared as the most likely topology (approximately unbiased test, AU: 1.00; bootstrap probability, NP: 1.000). The other two topologies, i.e. ‘Osteoglossomorpha sister to remaining teleosts’ and ‘Elopomorpha sister to remaining teleosts’ were rejected significantly (p-values < 0.00003 for all tests; see Fig. 3A and Supplementary Table 11). Thus, phylogenomic analysis and topology testing strongly supported the monophyletic clade of Elopomorpha and Osteoglossomorpha.

To further verify this relationship, we obtained transcriptome data from an additional elopomorph (*Megalops cyprinoides*, Indo-Pacific tarpon) and two osteoglossomorphs (*Pantodon buchholzi*, African butterflyfish; *Papyrocranus afer*, reticulate knifefish). We then generated ML and BI trees using 418 one-to-one orthologues from 12 species. Both ML and BI analyses gave the same topology as the 9-species dataset with strong support values (BS 100, PP 1.0; Fig. 3B). Topology testing also picked the “Elopomorpha sister to Osteoglossomorpha” arrangement as the most likely topology (AU: 1.00, NP: 0.999; Supplementary Table 12), and significantly rejected the other two alternate topologies (p-values < 0.002 for all tests; Supplementary Table 12).

The branching of these three groups is a typical case of near-simultaneous emergence of lineages that happened within a narrow temporal window of approximately 13 million years during the Early Permian^8^,^24^. Divergence events that occur during such short time spans result in reduced phylogenetic signal as there is not sufficient time for accumulation of a large number of synapomorphies. This is possibly why conflicting topologies have been obtained in previous molecular phylogenetic studies using smaller datasets. Since we used a genome-scale dataset, our data contains substantially larger number of phylogenetically informative characters. This might have helped in inferring the correct relationships. We note that previous studies have not identified any morphological synapomorphies between Osteoglossomorpha and Elopomorpha groups. Thus, there is a need to look for additional morphological characters that can be used to define these major teleost clades as well as to generate phylogenetic trees using independent data sets such as intron changes and other genomic markers.

We estimated the divergence times of spotted gar, arowanas and other teleosts using MCMCTree^25^ on 1,669,048 four-folds-degenerated sites extracted from 2,346 one-to-one orthologues together with three fossil calibration points. Our estimates showed that spotted gar and arowanas (teleosts) diverged 384 million years ago, which is consistent with estimates from a previous study^26^. Interestingly, our analysis also showed that the three colour varieties of arowanas diverged around 1 to 4 million years ago and have since been evolving as independent lineages (Supplementary Fig. 6).

### Hox gene clusters

Hox genes are attractive candidates for understanding the genetic basis of morphological diversity in animals as they play critical roles in defining identity of body segments^27^,^28^. Several studies have highlighted the crucial roles of Hox genes in establishing the body plan of teleosts. For example, duplication of Hox clusters and the resultant divergence of Hox gene expression pattern in the pufferfish are associated with the lack of ribs and pelvic fins^29^. In the Japanese flounder, it was proposed that *hoxb5* functions in the regional identification of gill arch 5, which in most teleosts has gained a masticatory function and is morphologically distinct from gill arches 1–4^30^. Previous analysis of Hox clusters in an osteoglossomorph, the African butterfly fish (*Pantodon buchholzi*)^31^, had shown that it contains only five Hox clusters (with 45 Hox genes), thus indicating that its lineage has lost three Hox clusters post-TGD. However, analysis of the whole-genome of the arowana indicated that it has retained all the eight Hox clusters following TGD similar to the European eel, a member of Elopomorpha^32^. The eight Hox clusters of arowana contain 59 Hox genes (Supplementary Fig. 7 and Supplementary Table 13), compared to 73 Hox genes in the European eel^32^. This finding suggests that the common ancestor of osteoglossomorphs contained a complete post-TGD set of eight Hox clusters and the African butterfly fish lineage subsequently lost three duplicate Hox clusters after splitting from the arowana lineage. Interestingly, although the arowana possesses more clusters than the African butterfly fish, it has lost some Hox genes that are retained in the butterfly fish. For example, the butterfly fish possesses duplicate copies of *hoxb4* (*hoxb4x* and *hoxb4y*), whereas the arowana retains only *hoxb4b* (Supplementary Fig. 7). Additionally, the arowana has lost both copies of *hoxd13* whereas the single HoxD cluster in butterfly fish has retained a *hoxd13* gene (*hoxd13x*) (Supplementary Fig. 7). These findings indicate that the complement of Hox genes can vary dramatically even between closely related lineages of vertebrates. Such variation in gene complement is characteristic of teleosts which have experienced an additional round of genome duplication followed by rapid differential loss of duplicate genes in different lineages^33^,^34^. The contribution of this Hox gene complement variation to the phenotypic differences between arowana and African butterfly fish remain to be determined.

### Analysis of differentially expressed genes in the three arowana colour varieties

In order to detect the potential molecular mechanisms underlying colour variation, we analyzed gene expression patterns in the combined scale and skin samples of the three arowana varieties (Supplementary Fig. 8). After comparing the normalized expression values of 18,178 “1:1:1” orthologous genes in the three arowana colour varieties, we identified 260 differentially expressed genes (DEGs; 2-folds, P-value < 0.05), which were then clustered into 10 groups according to their expression trends (Supplementary Fig. 9 and Supplementary Table 14). Intriguingly, we found that the golden and green varieties clustered together in a common branch based on their similar expression trends as compared to the red variety. This suggests that the genetic pathways of golden and green colorations share more genes and genetic networks than the pathway leading to red colouration (Supplementary Fig. 9). Specifically, we observed that the a- and b-paralogs of the four and a half LIM domains protein 2 (*fhl2a* and *fhl2b)* were significantly over-expressed (over 8-folds and P-value < 0.05) in the golden variety compared to the other two varieties (in group 7 of Supplementary Fig. 9 and Supplementary Table 14). The RNASeq-based expression patterns of *fhl2a* and *fhl2b* were confirmed by real-time PCR (Supplementary Fig. 10). Both *fhl2* paralogs, especially *fhl2b*, have been shown by previous studies^35^,^36^,^37^ to be involved in the formation of the egg-spot phenotype related to the production and deployment of xanthophores. It is possible that their high expression levels in the golden arowana could have contributed to the formation of increased number of xanthophores that in turn help to maintain the bright yellow colour in their scales and skin tissues.

### Chromosomal rearrangements in teleost lineages

**Figure 4:**
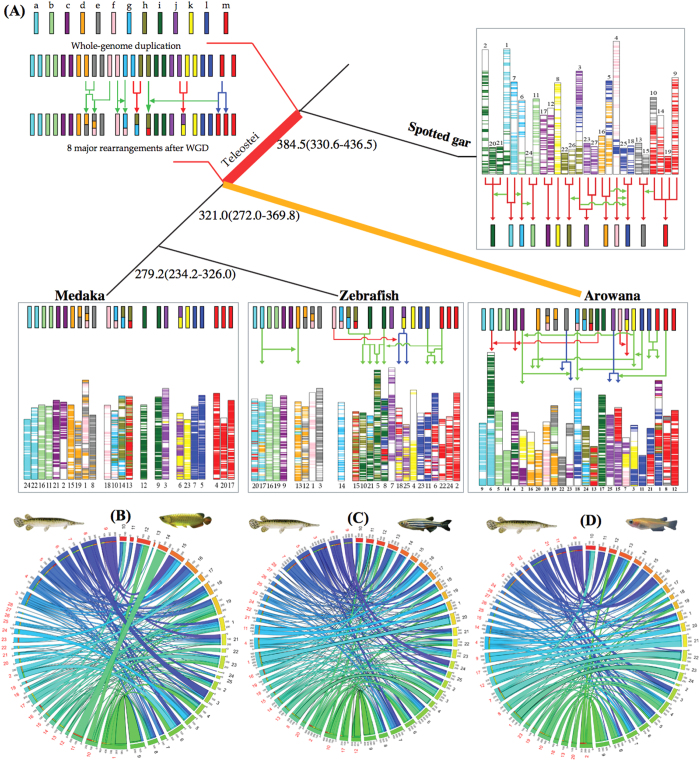
Evolution of the teleost karyotype. (**A**) Thirteen pre-TGD reduced ancestral chromosomes are indicated as coloured bars. Genomic regions originating from the same ancestral chromosomes are depicted in the same colour. Green, red and blue arrows represent translocation, fusion and fission events, respectively. The numbers in each branch of tree are the estimated divergence times. The predicted ancestral chromosomes of medaka were modified from Kasahara’s study^38^. Circos plots show syntenic relationships between the linkage groups of spotted gar and chromosomes of arowana (**B**) zebrafish (**C**) and medaka (**D**). Spotted gar chromosome numbers are shown in red whereas those of arowana, zebrafish and medaka are shown in black.

Reconstruction of the ancestral teleost karyotype and models of teleost genome evolution have been previously proposed based on genomes of clupeocephalan teleosts such as *Tetraodon* and medaka^4^,^38^,^39^. We aligned the arowana and human genomes, and identified 61 doubly conserved synteny (DCS, Supplementary Table 15) blocks, which is consistent with previous studies^4^,^38^. Comparison of the teleost ancestor chromosomes (from ref. 38) with those of golden arowana, revealed 12 major rearrangements comprising two fissions, three fusions and seven translocation events specific to the arowana (and possibly the whole Osteoglossomorph) lineage (Fig. 4A).

To further highlight the extent of rearrangements that occurred in different teleost lineages post-TGD, we analyzed inter-chromosomal rearrangements in the genome of arowana, zebrafish and medaka in detail using spotted gar as the reference. It has been proposed that the TGD event led to an increase in the rearrangement rates in various teleost lineages^40^. Our analyses revealed that the three teleost lineages have experienced different levels of inter-chromosomal rearrangements. The arowana genome shows a lower level of inter-chromosomal rearrangements (133) compared to zebrafish (179) but higher than that in medaka (116) (Fig. 4B–D, Supplementary Fig. 11 and Supplementary Table 20). The increased number of inter-chromosomal rearrangements in zebrafish may be related to the extremely high repeat content of its genome (52%)^41^ compared to arowana (27%, this study) and medaka (17.5%)^38^.

### Potential heterogametic sex chromosome in the female arowana karyotype

**Figure 5:**
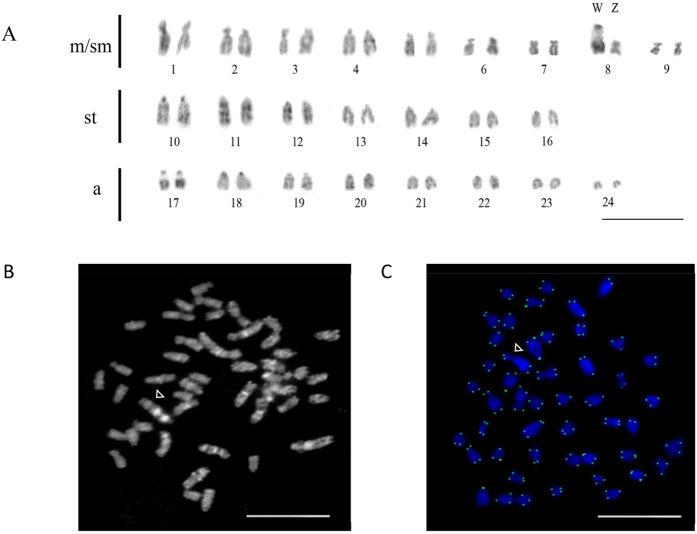
The karyotype of female Asian arowana contains a large, putative W chromosome. (**A**) A typical female karyotype showing 24 pairs of chromosomes, among them a pair with unequal sized chromosomes. A large acrocentric chromosome with a substantial heterochromatic block in the pericentromere region was identified as a putative W chromosome. Chromosome pairs 1–9 are metacentric/submetacentric (m/sm), 10–16 are subtelocentric (st) and 17–24 are acrocentric (a). (**B**) Chromomycin A3 staining. White arrowhead indicates the female-specific, putative W chromosome. (**C**) Metaphase chromosomes stained with DAPI (blue) and telomere probe (green). Bars are 5 μm for all panels.

We performed comparative analysis on the karyotype of six male and six female arowana individuals (Supplementary Tables 21 and 22). The results confirmed our earlier finding that the diploid chromosome number of arowana is 2 n = 48 (ref. 18). When the female karyotypes were compared to those of males (Fig. 5A), a large acrocentric chromosome with a substantial heterochromatic block in the pericentromeric region was identified (Fig. 5A) that showed the presence of a GC-rich region (Fig. 5B). This extra chromosome was present in all six females, but absent from the six males. The golden arowana reference genome was constructed in 25 linkage groups based on the RAD map (this study), which showed an apparent discrepancy with that of our earlier publication^18^ that reported 2 n = 48 as the male karyotype. We believe that this is due to the fact that the female assembly contains two differentiated sex chromosomes as separate units, thus resulting in an apparent increase of the haploid number of chromosomes.

Telomere signals were detected only at the ends of the chromosomes (Fig. 5C). FISH with 5 S and 18 S rDNA probes produced three signals on the karyotype of females compared to two in males (Supplementary Fig. 12). When the diploid copy number of the above two rDNA genes was examined by qPCR, the results indicated multiple copies of 5 S and 18 S rDNA loci, with the former being higher than the latter. Moreover, there was a difference between the 5 S rDNA copy numbers between the two sexes with the females showing a higher value (Supplementary Fig. 13; p < 0.05). The identification of a potential heterogametic sex chromosome in the female arowana karyotype suggests a ZW/ZZ sex chromosomal system.

We then used probes to identify the putative heterogametic sex chromosome and obtained five chromosomal fragments from the arowana karyotype through laser–based microdissection: two from the large, putative female-specific chromosome (AroW1 & AroW3), and three from autosomes (AroA1, AroA2 & AroA4) to be used as controls. The microdissected fragments were validated by hybridizing to female metaphase chromosomes using FISH. The results confirmed their origin, although signs of potential cross-hybridization on other chromosomes were seen for both putative W-specific probes and the autosomal ones (Supplementary Fig. 14A–C). MiSeq-based sequencing of the amplified microdissected chromosome fragments yielded the following number of clean reads: AroW1-291,124; AroW3-259,076; AroA1-377,429; AroA2-223,848 and AroA4-320,948. Sequences from these five read sets were mapped onto the 25 chromosomes of golden arowana reference genome. From every set, the majority of reads mapped to one or two chromosomes (designated as ‘source chromosomes’) and the rest were dispersed to several other chromosomes of the reference genome (Supplementary Table 23). Reads from the female-specific probes (AroW1 and AroW3) predominantly favoured Chromosome 4 (Chr4; 46.3%) followed by Chr2 and Chr17 (22.5% and 6.6%, respectively). The majority of autosomal fragment reads from AroA2 (78.6%) mapped to Chr 12, whereas those for AroA1 and AroA4, mapped to two (Chr23 – 36% and Chr9-33.3%) and three (Chr3-30.8%, Chr1-20.6% and Chr22-12.7%) chromosomes, respectively (Supplementary Table 23). The mapping of reads to multiple chromosomes was in agreement with the cross-hybridizations observed in the FISH analysis (Supplementary Fig. 14).

Next, all the successfully mapped reads were chained together across gaps less than 30 kb to form pseudo-scaffolds. The cumulative lengths of pseudo-scaffolds from the five sets were 37.3 Mb (AroW1), 8.4 Mb (AroW3), 0.99 Mb (AroA1), 14.8 Mb (AroA2) and 10.4 Mb (AroA4). The total number of genes from the four longest pseudo-scaffold sets ranged from 427 (AroW1) to 220 (AroA2; Supplementary Table 25). Unfortunately, comparative analysis of the repeat content and gene density on the four pseudo-scaffold sets did not yield additional indications towards the identity of the sex chromosome(s) (Supplementary Table 24).

We have also performed a cross-species comparison by mapping the Z and W chromosome-derived scaffold sequences from the tongue sole (which has a ZW/ZZ sex determination system^42^) to the 25 golden arowana chromosomes. The results showed that Chr17 the same chromosome that was among the three preferred targets of the putative sex-chromosome sequences earlier - had the largest aligned region (89% and 40%) in both cases, with Chr6-the biggest chromosome in the assembly-being the second and third largest region (53% [Z] and 19% [W], respectively; Supplementary Fig. 15). According to the evolution of the arowana karyotype (Fig. 4A) Chr2&4 and Chr6&17 both originated from a single ancestral chromosome, ‘c’ and ‘i’, respectively. Our knowledge about the evolution of sex determining (SD) systems and sex chromosomes in teleosts is quite limited, as the number of species with known SD is less than 20 and those with sequenced genome is less than 50 (Sridatta, P.S.R., personal communication) with limited overlap between the two sets. The data indicate high level of variation, as cross-species conservation of the SD could only be observed within the salmonids (13/15 species tested)^43^ and medakas (three species out of 14 tested from the *Oryzias* genus)^44^. As the tongue sole and the Asian arowana are evolutionarily distantly related, the potential functional overlap of the implicated homologous chromosomes would need to be investigated further.

Genetic and morphological degeneration followed by shrinking of the heterogametic sex chromosome (Y or W) has been observed in mammals, birds and snakes^45^,^46^. According to the universally accepted ‘addition-attrition hypothesis^47^ during the course of evolution, the heterogametic sex chromosome gradually loses its ability to recombine with its homogametic partner and starts to accumulate mutations resulting in a higher proportion of heterochromatin and fewer genes on the long term. In mammals (or birds) the size of the Y (or W) will typically shrink due to the above reasons. However, in the current study, an elongated W chromosome was observed in the karyotype of arowana females. A similar phenomenon was reported earlier in other fish species and plants, including tongue sole^42^, *Leporinus reinhardti*^48^ and papaya^49^. The apparent size increase of these W chromosomes could either be due to early accumulation of repeats that was shown to precede the transposon-driven decrease of size in heterogametic sex chromosomes^50^ or to a higher proportion of insertion to deletions or to a recent translocation of an autosomal fragment onto the proto-W chromosome^47^. In order to answer this question, genomes from both sexes need to be re-sequenced, and compared to the reference genome reported here (female; ZW).

A comparative analysis of the testis and ovary transcriptome was performed by using RNA-seq (Supplementary Table 25). Based on the GO term and Swiss-Prot results, the DEGs located on Chr2 (up-regulated in testis – 97; up-regulated in ovary – 63), Chr4 (106; 56), and Chr17 (134; 86) (Supplementary Table 25) were examined to identify those with potentially sex-related function. On Chr17, which had the largest aligned region (89%) with the tongue sole Z scaffolds, several genes with pro-male role in other species (e.g. *dmrt1, dmrt2, dmrt3a* and *piwl1*) were detected. On Chr2 and Chr4, genes with a pro-female role, such as *zp3* and *rabl3*, were identified, respectively. Validation was performed by quantitative PCR (qPCR) for 12 selected differentially expressed genes (DEG) (Supplementary Table 22) with high level of significance located on the three potential sex chromosomes and also three sex candidate genes *fhl3, dmrt3a* and *sf1* located on Chr5, Chr16 and Chr23, respectively. The data from the qRT-PCR analysis have successfully validated all of the 15 selected genes (Supplementary Fig. 16). Among the potential three sex candidate genes, *fhl3* showed the greatest level of differential expression in ovary versus testis, followed by *dmrt3* and *sf1*.

## Conclusions

In summary, we report a high quality chromosome-level reference genome of the golden Asian arowana, and draft genome assemblies of red and green varieties. Using phylogenomic analyses, we show that Osteoglossomorpha and Elopomorpha are a monophyletic group and thus resolve a long-standing controversy regarding phylogenetic relationships of the early branching lineages of teleosts. An unexpected finding is that arowana possesses all the eight post-TGD Hox clusters in contrast to another osteoglossomorph, the African butterfly fish, which has retained only five Hox clusters. This is a typical example of a whole-genome duplication event followed by extensive evolutionary changes to the gene complement in different teleost lineages. Such changes not only affect the gene number but can also alter the associated regulatory network and therefore have potential for giving rise to extensive phenotypic differences between closely related species. Comparative analysis of genes expressed in the skin and scales of three colour varieties provided insights into the genetic basis of colour variation, and methylation sequencing and gene knock-down experiments can further confirm the effect of these genetic variations. The genomic sequence combined with karyotype analysis identified putative sex chromosomes and suggest a ZW/ZZ sex determination system in Asian arowana.

Currently, the important unresolved problems of Asian arowana research include: 1) the lack of knowledge about the genetic diversity of most natural populations; 2) the relationship of color variants and new isolates that show morphological differences (see e.g. ref. 51); and 3) lack of understanding of the breeding biology of the species, especially partner selection and the mouth-brooding process. The data and resources generated in this study will be valuable for studies aiming to answer some of these questions.

## Materials and Methods

### Sample preparation for genome sequencing and RAD map generation

Second filial generation (F2) individuals of the golden variety arowana (2 year old, Tag number: 1828112203; CITES registration No. A-MY-508), the red one (1 year old, Tag number: 1828112146; CITES registration No. A-MY-508) and the green one (1 year old, Tag number: 1828112273; CITES registration No. A-MY-508) were collected from Pearl River Fisheries Research Institute, Chinese Academy of Fishery Sciences, where the fishes were introduced from Kim Kang Aquaculture Sdn. Bhd. of Malaysia in 2009.

Earlier, the Qian Hu fish farm (Singapore) obtained F1 hybrid individuals that originated from crossing two unrelated and genetically divergent founder (F0) Asian arowana grandparents (Red grade 1 x Malaysian golden varieties). Previously, we have generated two mapping families by crossing two pairs of these F1 hybrid brooders. The F2 offspring from these crosses were used for the generation of the first generation of genetic linkage map of the Asian arowana^18^. Here, a total of 94 offspring individuals from one mapping family and their parents were used for construction of the RAD map.

All animal experiments performed in China were in accordance with the guidelines of the Animal Ethics Committee and were approved by the Institutional Review Board on Bioethics and Biosafety of BGI. Animal experiments performed in Singapore at Temasek Life Sciences Laboratory were approved by Temasek Life Sciences Laboratory Institutional Animal Care and Use Committee (approval ID: TLL(F)-10-003) and performed according to its guidelines.

### Library construction, sequencing and filtering

Genomic DNA of golden, red and green arowana varieties were respectively extracted from several mixed tissues (including muscle, skin and liver) of a single individual per variety by the standard molecular biology techniques. We constructed the short-insert libraries (170, 500 and 800 bp for golden, 250 and 500 bp for red and green arowanas, respectively) and long-insert libraries (2 kb, 5 kb, 10 kb, 20 kb and 40 kb for golden variety, whereas 2 kb and 5 kb for both red and green varieties) with the standard protocol provided by Illumina (San Diego, USA). Paired-end sequencing with whole genome shotgun sequencing (WGS) strategy was performed using the Illumina HiSeq 2000 platform. We generated a total of 113.1, 103.8 and 90.5 gigabases (Gb) (Supplementary Table 1) of original reads from each library of golden, red and green varieties, respectively.

To improve the quality of sequence reads, we performed the following series of stringent filtering steps: 1) Discarded the reads with low-quality values (<20) or with 10 Ns (no sequenced bases); 2) Trimmed 6 and 5 bases of reads of short-insert and long-insert libraries; and 3) Filtered out the duplicated reads produced by PCR. Finally, we generated approximately 74.07 Gb (golden variety), 75.60 Gb (red variety) and 60.40 Gb (green variety) of clean reads (Supplementary Table 2) for the size prediction and assembly of genomes.

### Genome size prediction, sequence assembly and evaluation

#### Genome size estimation

The genome sizes of three varieties were estimated by the k-mer analysis with the formula G = N*(L-17 + 1)/K_depth, where N is the total number of reads, and K_depth indicated the frequency occurring more frequently than the others (Supplementary Fig. 1). We then calculated their genome sizes as 0.822 Gb (golden arowana), 0.949 Gb (red) and 0.897 Gb (green), respectively.

#### Genome assembly process

We employed the SOAPdenovo2^16^ (http://soap.genomics.org.cn/, version 2.04.4) software with optimized parameters trained artificially (pregraph -K 25 -d 1; contig -M 1; scaff -F -b 1.5 -p 16) to link the sequenced reads to contigs and original scaffolds. All reads were then aligned onto the contigs for scaffold construction by utilizing the long-insert paired-end information. This paired-end information was subsequently supplied to link contigs to scaffolds step by step. Some intra-scaffold gaps were filled by local assembly software using the reads in a read-pair where one end uniquely mapped to a contig whereas the other end was located within a gap.

#### Transcriptome evaluation for genome assemblies

This analysis aimed to assess the completeness of gene regions in genome assembly. We firstly *de novo* assembled the RNA sequences of skin and scale tissues of three arowana varieties by using the Trinity software^52^. The assembled fragments were aligned to genome assemblies with BLAT^53^ (E-value = 10e^−6^, identity = 90% and coverage >90%). The results indicated that the genomes of golden, red and green arowanas covered over 90% of the gene coding-regions (Supplementary Table 4).

#### CEGMA assessment of genome assemblies

CEGMA software^54^ (Core Eukaryotic Genes Mapping Approach) (http://korflab.ucdavis.edu/Datasets/genome_completeness, version 2.3) with 248 conserved Core Eukaryotic Genes (CEGs) was employed to assess the gene space completeness of the three genomes. The results showed that all three assemblies covered more than 95% of the CEG sequences, indicating their high level completeness (Supplementary Table 5).

### RAD sequencing and linkage group construction

#### RAD Sequencing

Genomic DNA was isolated from the scales and/or fin clips of all the offspring individuals and their parents of the F2 mapping family by using Mag Attract HMW DNA Kit. The DNA was then digested with the restriction endonuclease EcoRI and processed into 3 RAD libraries^55^. A total of 72.8-Gb reads with 101-bp length (evenly 800 Mb of raw data for each individual) were sequenced by the HiSeq 2000 platform.

#### RAD SNP calling

After filtering the adapters and removing low quality reads, we mapped the filtered reads onto the golden assembly (reference) by using SOAP2 (version 2.21)^56^ software with common parameters (-m 100 –x 888 –s 35 –l 32 -v 3 -p 4). Then we employed the Samtools^57^ to identify the single nucleotide polymorphisms (SNPs) in each individual. We have discarded the SNPs whose missing rates were higher than 30%, and we utilized the χ2 test to confirm whether the RAD-based SNP markers were consistent with the expected segregation ratio. This way, we removed the markers whose P-value were lower than 0.01.

#### Genetic map clustering

Linkage group clustering and linkage distance calculation were performed by Joinmap 4.1^58^ with optimized parameters: Chain length per Monte Carlo EM cycle = 1000, Sampling period for rec. freq. matrix sample = 5, Chain length = 1000, Initial acceptance probability = 0.250, Cooling control parameter = 0.00100, Stop after # chains without improvement = 10000, Length of burn-in chain = 10000 and Nr. Of Monte Carlo EM cycle = 4. All the selected SNPs were clustered in 25 linkage groups, which were in agreement with the previous chromosome karyotype of arowana (2n = 48 and one additional W chromosome)^18^. Ultimately, 87.65% scaffolds (683.04 Mb/779.26Mb) were anchored onto 25 linkage groups, and the detailed statistics of chromosome length, marker number, genetic distance and physical length were shown in Supplementary Table 6 and Supplementary Fig. 2.

### Annotation of repetitive sequences and protein-coding genes and functional assignments

#### Repeat annotation

First, we used the RepeatModeller (http://www.repeatmasker.org/RepeatModeler.html, version 1.04) and LTR_FINDER^59^ to build a *de novo* repeat library with default parameters, and then utilized the RepeatMasker^60^ (http://www.repeatmasker.org/, version 3.2.9) to align our sequences against the Repbase^61^ TE (version 14.04) and the *de novo* repeat libraries to search for known and novel transposable elements (TEs). Next, we also annotated the tandem repeats by using the Tandem Repeat Finder^62^ (http://tandem.bu.edu/trf/trf.html, version 4.04) with major parameters set as “Match = 2, Mismatch = 7, Delta = 7, PM = 80, PI = 10, Minscore = 50, and MaxPerid = 2000”. Furthermore, the TE relevant proteins were identified in our assemblies by using the RepeatProteinMask software (http://www.repeatmasker.org/, Version 3.2.2).

#### Gene structure and function annotation

The genome assemblies of three arowana varieties were annotated by three independent pipelines containing homology, *de novo* and RNA-seq annotations: 1) Homology annotation: The protein sequences of *H.sapiens* (human), *D.rerio* (zebrafish), *T.rubripes* (Japanese fugu), *T.nigroviridis* (spotted green pufferfish), *G.aculeatus* (three-spined stickleback), *O.latipes* (Japanese medaka) proteins, *Cynoglossus semilaevis* (tongue sole) and *Latimeria chalumnae* (coelacanth) (Ensembl release 75) were downloaded and aligned to the genomes of golden, red and green arowana varieties using TblastN with e-value ≤ 1E-5. Then we analyzed the data with Genewise2.2.0^63^ software to predict the potential gene structures on all alignments. The short genes (less than 150 bp), and prematurely terminated or frame-shifted genes were discarded. 2) *De novo* annotation: At first, we randomly selected 1,500 complete genes from the results of homology annotation set to train the parameters for AUGUSTUS2.5^64^. Simultaneously, we masked all the repetitive regions to be “N” in the three genomes to prevent the pseudogene annotation. Subsequently, we utilized AUGUSTUS2.5^64^ and GENSCAN1.0^65^ for *de novo* prediction on repeat-masked genome sequences. The filtered processes performed on the *de novo* annotation were the same as the one used for homology prediction. 3) RNA-seq annotation: We employed the Tophat1.2^66^ software to map the RNA reads extracted from the skin and scale tissue of golden, red and green arowana varieties onto their genome sequences, respectively. We then sorted and integrated the alignments of Tophat where we used the Cufflink (http://cufflinks.cbcb.umd.edu/) software to search possible gene structures. All results from above three annotation pipelines were merged to produce a comprehensive and non-redundant gene set using GLEAN^67^. The Cuffdiff package^68^ of Cufflink software (version 2.0.2.Linux_x86_64) with core parameters (–FDR 0.05 –geometric-norm TRUE –compatible-hits-norm TRUE) was utilized to calculate expression level based on the GLEAN gene set and Tophat alignments. Simultaneously, all protein sequences from the GLEAN results were aligned to SwissProt and TrEMBL^19^ (Uniprot release 2011.06) by BlastP with an E-value 1e-5 to find the best hit for each protein. We also used the InterProScan4.7^69^ software to align the protein sequences against the public available databases including Pfam, PRINTS, ProDom and SMART for examining the known motifs and domains in our sequences of the three variety arowanas. Finally, we filtered the GLEAN gene set in three steps to remove: 1) gene with their length is shorter than 150 bp; 2) genes identified as TEs; and 3) genes that were only generated from *de novo* pipeline and with an expression value (FPKM: Fragments Per Kilobase of exon per Million fragments mapped) lower than 1 without functional assignment. This process yielded the refined gene sets that contained 22,016 genes (golden), 21,256 (red) and 21,524 (green; Supplementary Table 8). Over 70% of the genes were annotated by all the three pipelines, approximately 95% of the genes were predicted from at least two types of evidences (Supplementary Fig. 3), and approximately 87% of the genes showed expression activity (FPKM > 0) (Supplementary Table 9). On the other hand, over 96% of these genes (Supplementary Table 10) from three arowana varieties possess at least one related functional assignments from the public databases (Swiss-Prot, Interpro, TrEMBL and KEGG). In addition, the gene structures (including the length distributions of exon, coding regions and mRNA) and exon number distribution of three varieties were consistent with other representative fish species, like zebrafish and medaka (Supplementary Fig. S4). All above statistical numbers show that our gene sets of the three arowana varieties are indeed of high quality.

### Determining the phylogenetic position of Asian arowana

#### Ortholog identification and extraction

In order to determine the phylogenetic position of Asian arowana (*Scleropages formosus*, Osteoglossomorpha) and the branching order of the three major clades of Teleostei (Clupeocephala, Osteoglossomorpha and Elopomorpha), we performed phylogenomic analyses with orthologues from representative species for each clade. We used the Ensembl BioMart (www.ensembl.org/biomart; Ensembl version 76) to extract orthologues for zebrafish, fugu, stickleback, medaka, spotted gar and coelacanth. This six species orthologue dataset was filtered out to retain only one-to-one orthologues. The resultant six-species one-to-one orthologue set contained 5,354 genes. We then downloaded the electric eel (Gymnotiformes, Clupeocephala; gene coordinates and genome assembly, http://efishgenomics.zoology.msu.edu) and European eel (Elopomorpha; http://www.zfgenomics.org/sub/eel) datasets. The Asian arowana gene set is from the present study. In order to extrapolate the Biomart orthologues to the arowana, European eel and electric eel gene sets, we used zebrafish as the reference. We ran InParanoid^70^ for the three species pairs (zebrafish-arowana, zebrafish-European eel and zebrafish-electric eel) at default settings (i.e., minimum 50% alignment span, minimum 25% alignment coverage, minimum BLASTP score of 40 bits, minimum inparalog confidence level of 0.05) in order to identify orthologues between the three pairs. By comparing the three InParanoid outputs with a list of 5,354 zebrafish genes from the BioMart dataset, we narrowed down the list of one-to-one orthologues present in all nine species. This nine-species one-to-one orthologue dataset comprised 2,463 genes (Dataset 1).

In order to improve taxon sampling for the groups Elopomorpha and Osteoglossomorpha, we generated transcriptome data for representative species – Indo-Pacific tarpon (*Megalops cyprinoides*, Elopomorpha), African butterflyfish (*Pantodon buchholzi*, Osteoglossomorpha) and reticulate knifefish (*Papyrocranus afer*, Osteoglossomorpha). The transcriptome data were assembled using SOAPdenovo-Trans^71^ and candidate coding regions within the transcript sequences were identified using TransDecoder (http://transdecoder.sourceforge.net/). Redundant sequences (identical or nearly identical) were removed by CD-HIT clustering^72^ using a threshold of 99% identity and 90% coverage (smaller sequence). To ensure that only full-length or near full-length proteins were used for orthologue identification, we searched these non-redundant proteins against a RefSeq database of full-length proteins from six organisms (zebrafish, fugu, medaka, stickleback, spotted gar and coelacanth). A cut-off of 1e-5 was used for BLASTP and only the top hit was considered. Proteins with ≥80% alignment coverage were considered as full-length proteins. The CD-HIT clustered, non-redundant transcriptome protein datasets from Indo-Pacific tarpon, African butterflyfish and reticulate knifefish were used for InParanoid^70^ against the zebrafish proteome to identify one-to-one orthologues. The InParanoid outputs were then compared with their respective full-length datasets to get a set containing full-length one-to-one orthologues from the tarpon, butterflyfish and knifefish. Zebrafish gene identifiers from the 9-species dataset (2,463 genes, Dataset 1) were used to filter this set. Finally, comparison of the filtered dataset from tarpon, butterflyfish and knifefish to the remaining 9-species identified a set of 418 one-to-one orthologues for the 12 species (Dataset 2).

#### Phylogenetic analyses using genome-scale datasets

Multiple alignments were generated at the protein level for each of the 2,463 (9 species, Dataset 1) or 418 (12 species, Dataset 2) one-to-one orthologues using ClustalW^73^. Coding sequence alignments were generated from respective protein alignments using PAL2NAL^74^. Concatenated nucleotide alignments were prepared for the 9- and 12-species alignments by merging the individual coding sequence alignments. The concatenated coding sequence alignments were used for phylogenomic analyses. Alignment gaps and ambiguous positions were removed using Gblocks version 0.91b^75^. The best-suited substitution model for each alignment was deduced using ModelGenerator version 0.85^76^. We used Maximum Likelihood (ML) and Bayesian Inference (BI) methods for phylogenetic analyses. ML and BI trees were generated using RAxML version 8.1.3^77^ and the parallel (MPI) version of MrBayes 3.2.3, respectively^78^,^79^. For the ML analyses, we used RAxML’s rapid bootstrapping algorithm plus a thorough ML search (-f a option) and 1000 bootstrap replicates for node support. For the BI analyses, two independent runs starting from different random trees were run for five million generations with sampling every 100 generations. A consensus tree was built from all sampled trees excluding the first 25% (12,500 samples) which were discarded as ‘burn-in’.

#### Testing of alternate tree topologies

We evaluated the likelihood of alternate tree topologies using CONSEL^80^. Site-wise log-likelihood values were generated for the topologies being tested using the “-f g” option implemented in RAxML version 8.1.3^77^. These values were used as an input to CONSEL. Only the following three topologies are possible for these teleost groups:

1. ((Clupeocephala, (Elopomorpha, Osteoglossomorpha)), outgroups)
2. (((Clupeocephala, Elopomorpha), Osteoglossomorpha), outgroups)
3. (((Clupeocephala, Osteoglossomorpha), Elopomorpha), outgroups)

We evaluated the likelihood of the tree topologies corresponding to these relationships for both Dataset 1 and Dataset 2. Topology 1 has been suggested in a single study that was based on partial sequences of the nuclear 28S ribosomal RNA gene^12^. Topology 2 is based on previous analyses of partial or complete mitogenomic sequences^11^,^81^,^82^. Topology 3 was suggested based on analyses of nuclear genes^8^,^13^,^24^,^83^ and ultraconserved elements^10^.

#### Estimation of divergence time

To estimate the divergence times among arowanas and other teleosts, the MCMCTree software from the PAML package^25^ was used to calculate the divergence time basing on 1,669,048 four-folds-degenerated sites extracted from 2,346 one-to-one genes with 3 calibration fossil records. The estimated results and used calibration fossil records were shown in Supplementary Fig. 6.

### Hox gene identification

Hox genes were predicted in the golden arowana genome assembly based on homology to known Hox genes. The predictions were manually inspected and refined. Sequencing gaps within the Hox clusters, particularly those between *hoxb5a* and *hoxb3a*, and *evx2* and *hoxd12a* were filled by PCR amplification and Sanger sequencing.

Phylogenetic analysis is generally considered a reliable approach to establish the orthology of the duplicate Hox cluster paralogs. However, in the case of the European eel and the African butterfly fish, phylogenetic analysis was uninformative in assigning the duplicate Hox clusters to the Clupeocephalan paralog clusters ‘a’ and ‘b’^31^,^32^. For the African butterfly fish, since the authors were unable to establish orthology relationships for the duplicate Hox clusters, the Hox clusters were named as hox-ax, -bx, -by, -cx and -dx clusters^31^. On the other hand, the whole-genome sequence of the European eel allowed the authors to analyze the synteny around the Hox clusters and to assign orthology based on unique patterns of syntenic genes flanking the Hox clusters^32^. Similar to the European eel, we used unique patterns of syntenic genes flanking the Hox clusters to assign them to the Clupeocephalan Hox paralog clusters ‘a’ and ‘b’ (see Supplementary Table 13). For example, the presence of genes *evx1* and *nfe2l* were used as a signature to distinguish the HoxAa cluster from the HoxAb cluster. Similarly, genes *mfsd5* and *spryd3* were used as a signature to classify the HoxC clusters as HoxCa and HoxCb, respectively. Using this approach, we could assign each of the arowana Hox cluster to the Clupeocephalan paralog copy ‘a’ or ‘b’ (Supplementary Table 13).

### Detection of differentially expressed genes in the skin and scale tissues of three arowana colour varieties

We collected scale and skin tissues of the three arowana individuals and sequenced their transcriptome with RNA-seq using the Hiseq2000 platform. We subsequently mapped the cleaned RNA reads to their corresponding genome assemblies by using Tophat software^66^. After obtaining the aligned results processed by sorting and merging, we utilized the Cufflink^68^ (version 2.0.2.Linux_x86_64) to calculate the FPKM of each sample (all the FPKM data was shown in Supplementary Table 9). At last, the edgeR software^84^ was used to identify and draw the significantly differentially expressed genes in three samples with the threshold: P-value < 0.05 and folds >2 (Supplementary Table 14 and Supplementary Fig. 10).

We then performed the real-time PCR to validate the expression values of *fhl2a* and *fhl2b* genes. Firstly, we collected the adult fin clips from three golden, three red and three green arowana individuals. Two pairs of primers (Fhl2a-Fs: AGCTTTCATGAGCCTCGGTA and Fhl2a-Rs: CCAGGCATGATGGTCTTTTT; Fhl2b-Fn: GCCAGATGAGAAGGTGGAGT and Fhl2b-Rn: GTTGTCTTTCGGGATGAAG; all in 5′ -3′ orientation) were designed to evaluate the transcription level of long and short transcripts of the *fhl2a* and *fhl2b* genes in nine samples. 18S RNA was amplified as an internal control. The PCR products were excised, purified and subcloned into the pMD19-T vector for sequencing to confirm whether it was the target sequence. Single-stranded cDNA was synthesized from 1 μg of total RNA from each sample using the PrimeScript1st Strand cDNA Synthesis Kit (TaKaRa, Dalian, China). Amplification of each sample was performed in triplicate with each reaction well containing 20 μL of a PCR mixture consisting of 1 μL cDNA template, 10 μL SYBR Premix (TaKaRa, Dalian, China), 0.6 μL forward and reverse primer (20 pmol/L), 0.4 μL ROX reference dye, and 8 μL dd H_2_O. The PCR reaction was performed using the ABI Stepone plus (Applied Biosystems, Foster City, CA, USA) with SYBR Premix Ex TaqTM (TaKaRa, Dalian, China). A melting curve analysis was performed over a range of 60–95 °C to confirm single product generation at the end of the assay. The relative expression level of the gene was calculated using the 2 ^−ΔΔCt^ method^85^ (Supplementary Fig. 11).

### Reconstruction of ancestral vertebrate chromosomes

First, we performed a two-way comparison between the protein sets of golden arowana and human by using the BLASTP (E-value < 1 × 10^−10^) to search the paralogs in the arowana genome (Supplementary Table 16). We then identified the double-conserved syntenies and then deduced the ancestral teleost karyotype by analysing the results from the human genome as an outgroup using the similar method from previous study^38^. In addition, we paired paralogous chromosomes according to the number of paralogs between two chromosomes. We collected spotted gar (release 75), medaka and zebrafish gene sequences from Ensembl (release 64) and identified reciprocal best-hit genes between golden arowana and each of three above indicated fish species using BLASTP (E-value of 1e^−10^). A total of 11,639, 10,846 and 12,103 orthologous genes (Supplementary Tables 17-19) were identified for spotted gar, medaka and zebrafish, respectively. Finally, the ancestral teleost karyotype was predicted to have 13 chromosomes, represented as Ancestor Chromosomes a~m that was indicated in previous study^38^. We then deduced the chromosome fission, fusion and translocation events by comparison with the recent chromosomes of arowana, spotted gar and zebrafish and ancestor chromosomes (Fig. 4A).

### Inter-chromosomal rearrangement events in teleost fish lineages

To identify major inter-chromosomal rearrangement events in arowana and other teleosts, we used spotted gar as an outgroup since it has not experienced the teleost-specific whole-genome duplication. We identified orthologues for the pairs spotted gar-arowana, spotted gar-zebrafish and spotted gar-medaka using InParanoid^86^. The orthologue gene sets were used to identify orthologous regions/syntenic blocks in the genome pairs using i-ADHoRe v3.0 129 (ref. 87). The following parameters were used: “alignment_method = gg4, anchor_points = 3, tandem_gap = 15, gap_size = 30, cluster_gap = 35, q_value = 0.75, prob_cutoff = 0.01, level_2_only = false”. The program first identifies homologous regions (segments) in two genomes that contain at least three homologous genes (anchorpoints) with the anchorpoints separated by at most 30 non-homologous genes (‘gap_size’). These form the base-clusters (with minimum quality factor of 0.75 and probability cut-off of 0.01), which are then grouped into larger syntenic blocks (‘multiplicons’) if they are within 35 genes (‘cluster_gap’) of each other. Considering only the non-redundant ‘multiplicons’ (syntenic blocks) and their corresponding ‘anchor points’ (homologous genes of the syntenic segments), syntenic blocks between the three genome pairs were identified, and the number of orthologous genes in the syntenic blocks was tabulated. Synteny plots for the three pairs were generated using the visualization tool Circos version 0.66 (ref. 88).

### Analysis of the sex chromosomes of Asian arowana

#### Sample collection

Tissue samples for karyotyping were collected from six adult male and six adult female Asian arowana hybrids at the Qian Hu Fish Farm. These fish were F1 offspring individuals produced by crossing chili red and Malaysian golden brooders. They have been pit-tagged upon maturation and used as brooders on a regular basis by the farm. Their sex has been confirmed through the analysis of sexually dimorphic morphometric traits. The arowanas were tranquilized by Tricane methane sulphonate prior to sample collection. Small pieces of fin clips and individual scales were removed from the belly area of ten males and ten females and stored on ice for less than two hours prior to use for DNA extraction and following copy number estimation of rDNA by using qPCR.

Tissue samples for RNAseq and qPCR-based validation were obtained from three male and three female adult Asian arowanas (sex was confirmed by dissection) of the golden variety from Qian Hu Fish Farm (Singapore). The tag numbers for all of 26 individuals are listed in Supplementary Table 21, whereas primers used for PCR are listed in Supplementary Table 22.

#### Primary culture and chromosome preparation

The fin clips and scales with a small piece of tissue from the scale pocket were seeded into cell culture dishes and plated in RPMI Medium 1640 (Life Technologies) supplemented with 20% fetal calf serum containing antibiotic and antimitotic solution (Sigma-Aldrich, USA). The primary cultures were incubated at 29 °C with 5% CO_2_. After 1–2 weeks, the cells were incubated with 0.01% of colchicine for 5–6 hours. Chromosome spreads were prepared using the method outlined earlier^89^. Images were acquired using Zeiss/MetaMorph epifluorescence microscope equipped with a (CCD) camera.

#### Karyotype production and genome size determination

In order to determine the karyotype of Asian arowana hybrids, ten good male and female metaphase plates from each of the six males and six females were used. The classification of chromosomes followed the method of Levan and colleagues^90^. Submetacentrics (SM) were described as two-arm chromosomes and acrocentrics (A) as one-arm chromosomes. The karyotype of Asian arowana hybrids appeared very similar to those of red female, golden male and golden female individuals of Asian arowana (data not shown). The relative nuclear DNA content of five male and five female hybrid Asian arowana individuals was determined according to the technique described by Carvalho and colleagues^91^ with minor modifications. Fresh and frozen livers were processed to obtain single-cell suspensions that were fixed in ethanol, stained with propidium iodide and analyzed by flow cytometry^18^,^92^. The genome size of Asian arowana was estimated by multiplying the genome size of chicken (standard) by the ratio of their fluorescent intensities (G0/G1 means).

#### Quantitative PCR analyses from genomic DNA

Genomic DNA was extracted from fin clips of 10 male and 10 female mature hybrid arowana individuals. 5S rDNA and 18S rDNA sequences were amplified from the genomic DNA. In order to quantify 5S rDNA and 18S rDNA levels, we used published ribosomal DNA-specific primers (Supplementary Table 22) designed to amplify a fragment from single-exon genes^93^,^94^. Quantitative PCR (gDNA-qPCR) was performed using MyiQ BioRad system, in 20-ul reaction volume with 10-ng template and the Power SYBR Green reagent (Applied Bioscience) according to the protocol recommended by the manufacturer (40 cycles, 60 °C). Each data point represents an average obtained from three qPCR reactions. The single-copy reference gene was gal3st3 (galactose-3-O-sulfotransferase 3: primers are listed in Supplementary Table 22). Melting curve analyses were performed following amplifications. Results are reported as mean + /− standard error. Statistical analysis of differences between Ct values was performed with the Student’s t-test. In all cases, a value of p < 0.05 was used to indicate significant differences.

#### Chromosome microdissection, amplification and testing by Fluorescent in Situ Hynridization (FISH)

Chromosome-specific probes were generated from a red x golden hybrid female Asian arowana individual as described previously^95^. The morphology of chromosomes was determined based on Giemsa staining (Merck, USA). Chromosomes were collected using a glass needle coupled with an inverted microscope (Olympus, Germany) and placed into collection drop solution (30% glycerol, 10 mM Tris/HCl, pH 7.5, 10 mM NaCl, 0.1% SDS, 1 mM EDTA, 0.1% Triton X-100, 1.44 mg/L proteinase K), and incubated at 60 °C for an hour. Primary PCR products were used for the preparation of detection probes (for FISH experiments) and library preparation for sequencing.

Probes for 5S rDNA and 18S rDNA delete and replace with (Supplementary Table 22) were synthesized as described earlier^96^, whereas the telomere probe was obtained by PCR-amplification with a (TTAGG)_5_ primer. Probes were labelled with digoxigenin-11-dUTP (DIG) for FISH. The labeled nucleotides were incorporated into fragments by PCR.

The specificity of the microdissected material was tested by FISH. An initial round of chromosomal DNA amplification was performed using the WGA 1 Kit (Sigma-Aldrich) with the following protocol: 16 °C for 20 minutes, 24 °C for 20 minutes, 37 °C for 20 minutes, then 75 °C for 5 minutes. The WGA-PCR-amplified chromosomal material was re-amplified with 16-dUTP-biotin and digoxigenin-11-dUTP (both 2 μM, Roche) under the following conditions: (1×) 94 °C for 5 min; (35×) 90 °C for 30 sec, 54 °C for 30 sec, 72 °C for 30 sec using the WGA3 reamplification kit (Sigma-Aldrich) and used as painting chromosomes-specific probe in FISH experiments.

Chromosomes were denatured in 70% deionized formamide, 2 x SSC at 72 °C for 2 min and dehydrated in an ethanol series. A DNA mixture of approximately 400 ng of the chromosome painting probe and 10 μg of the Cot-1 fraction of the Asian arowana DNA was prepared by treatment with S1-nuclease (Sigma-Aldrich), ethanol precipitated, dissolved in 20 μl of hybridization mixture containing 50% deionized formamide, 10% dextran sulphate, and 2 x SSC. After denaturation (10 min at 75 °C) the probe was incubated 30 min at 37 °C and then dropped onto a slide and spread over the hybridization area using glass coverslip. Slides were incubated for 24 h at 37 °C in a humid chamber.

Post-hybridization washes were in 4× SSC for 5 min at 73 °C and 2x SSC for 5 min at room temperature. After wash in the PBST (PBS, 0.1% Tween), slides were incubated with biotinylated anti-rhodamin (Vector Laboratories, USA) and FITC-conjugated anti-DIG antibody (Roche, USA). Finally, the slides were counterstained with DAPI and mounted in an antifade solution (Vectashield from Vector Laboratories). Images were captured with a Nikon (CCD) camera on a Zeiss/MetaMorph epifluorescence microscope for paint using Adobe Photoshop CS2.

#### Library construction, sequencing and post-processing of the sequences

Sequencing libraries from the microdissected chromosome fragments were prepared using the NEBNext DNA Library Prep Master Mix Set for Illumina (Illumina, USA). Libraries were sequenced on the Illumina MiSeq System with read length configuration of 2х250 bp.

We removed all reads with mean quality less than 20, trimmed the WGA-specific adapter sequences (TGTGTTGGGTGTGTTTGG) using the Cutadapt (Martin 2011) program, and trimmed low quality bases using “Trim Galore!” software (http://www.bioinformatics.babraham.ac.uk/projects/trim_galore/) with default parameters. We then cleaned out reads containing 23-mer Illumina-specific primers using Cookiecutter (https://pypi.python.org/pypi/cookiecutter/0.7.2).

The cleaned reads from the microdissected chromosomes were mapped to the three assembled Asian arowana genomes (golden, red and green) using Bowtie2 with default parameters in sensitive mode, separately. Successfully mapped reads were chained together across gaps less than 30 kb to form pseudo-scaffolds by the B-chromosomer tool (https://github.com/ad3002/B-chromosomer). Repeat-masking was performed using the de novo Asian arowana repeat database obtained by *de novo* with RepeatScout^97^ and with Repbase vertebrate-specific repeat library^61^. Gene descriptions were assigned to the chromosome pseudo-scaffolds based on the gene predictions of Asian arowana genomes and the annotation was lifted over from of the corresponding annotation gff3 file with in-house scripts.

In an attempt to identify the potential sex chromosomes, cleaned reads from the amplified, microdissected DNA were mapped to the chromosome-level golden arowana reference genome with 25 chromosomes (as the other two genome assemblies were only at pseudo-scaffold level). Secondly, the Z chromosome scaffold sequences of the tongue sole^42^ were compared to the same set of chromosomes using Symap 4.2 (Ref. 98). Further, based on gonadal RNASeq results, the differentially expressed gonadal genes on those potential sex chromosomes were identified.

#### Comparative analysis of gonad transcriptomes

Comparative transcriptome analysis between the testes and ovaries from three adult golden Asian arowana individuals each was conducted using RNA-seq data. The comparative transcriptome analysis identified a total of 4,264 transcripts that were differentially expressed between the two gonads, including 2,827 genes with up-regulated expression in testis and 1,437 up-regulated in ovary (Supplementary Table 26). Validation was performed by quantitative PCR (qPCR) for 15 selected genes (for primers see Supplementary Table 22). Total RNA was isolated from six adult Asian arowana gonads (three males and three females) by using miRCURY™ RNA Isolation Kit (Qiagen), and quantified using Qubit 2.0 and Bioanalyzer 2100 (Agilent, USA). The qPCR was performed on ABI PRISM 7900 Real-Time PCR System using KAPA SYBR Green PCR Kits. A total of 800 ng of RNA of each samples were reverse-transcribed by using iScriptTM Reverse Transcription Supermix (BIO-RAD, USA: Cat #170-8841). The 20 μL PCR reaction includes 10 μL of iTaqTM Universal SYBR® Green Supermix (BIO-RAD, Cat #172-5124), 0.8 μL of each primer (0.4 μM final concentration) and 2 μL of cDNA. Out of several reference genes tested, *rpl13a* gene was selected as the reference gene. Melting curve analyses were performed following amplifications. Quantification of the abundance of selected mRNA transcripts was performed based on PCR amplification efficiencies and crossing point (CT) differences^99^.

## Additional Information

**Data availability**: The whole-genome assemblies of golden, red and green varieties of Asian arowana were deposited in GenBank under project accession LGSG01000000, LGSF01000000 and LGSE01000000, respectively.

**How to cite this article**: Bian, C. *et al.* The Asian arowana (*Scleropages formosus*) genome provides new insights into the evolution of an early lineage of teleosts. *Sci. Rep.* **6**, 24501; doi: 10.1038/srep24501 (2016).

## Supplementary Material

Supplementary Dataset 1

Supplementary Dataset 2

Supplementary Dataset 3

Supplementary Information

## Figures and Tables

**Figure 1:**
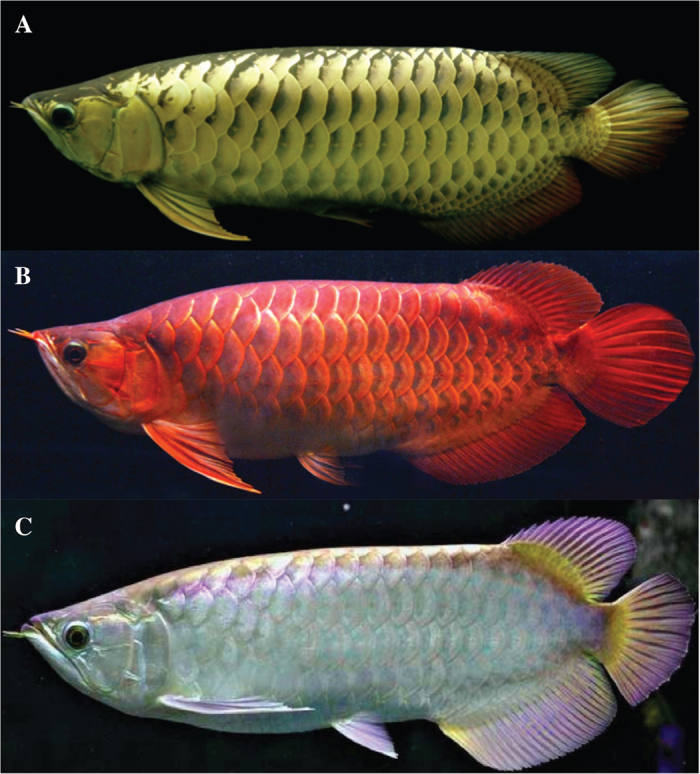
The three colour varieties of Asian arowana sequenced in this study. (**A**) golden, (**B**) red and (**C**) green variety. These are among the most expensive ornamental fishes in the world (young adults for red arowanas cost from $1500 to $2000). The value of the fish depends on the colour with the red variety fetching the highest price.

**Table 1:** Overview of the genome assembly and annotation for the three colour varieties of Asian arowana.

Colour variety	Golden	Red	Green
Sequence coverage (-fold)	138	110	100
Estimated genome size (Gb)	0.82	0.95	0.90
Assembled genome size (Gb)	0.78	0.75	0.76
Scaffold N50 (Mb)	5.97	1.63	1.85
Contig N50 (kb)	30.73	60.19	62.80
Number of genes	22,016	21,256	21,524
Repeat content	27%	28%	28%
