# Supplementary material for: The Asian arowana (Scleropages formosus) genome provides new insights into the evolution of an early lineage of teleosts
Source: Sci Rep. 2016 Apr 19;6:24501. doi: 10.1038/srep24501 (PMC4835728; doi:10.1038/srep24501)
Supplement: Supplementary Information [file srep24501-s4.doc]

**The Asian arowana *(Scleropages formosus)* genome provides new insights into the evolution of an early lineage of teleosts**

Chao Bian^1,2^*, Yinchang Hu^3^*, Vydianathan Ravi^4^*, Inna S. Kuznetsova^5,6^*, Xueyan Shen^5^*, Xidong Mu ^3^*, Ying Sun^2^*, Xinxin You^1,2^, Jia Li^1,2^, Xiaofeng Li^7^, Ying Qiu^1,2^, Boon-Hui Tay^4^, Natascha May Thevasagayam^5^, Aleksey S. Komissarov^8^, Vladimir Trifonov^9,10^, Marsel Kabilov^11^ , Alexey Tupikin^11^, Jianren Luo^3^, Yi Liu^3^, Hongmei Song^3^, Chao Liu^3^, Xuejie Wang^3^, Dangen Gu^3^, Yexin Yang^3^, Wujiao Li^2^, Gianluca Polgar^12^, Guangyi Fan^2^, Peng Zeng^2^, He Zhang^2^, Zijun Xiong^2^, Zhujing Tang^2^, Chao Peng^1,2^, Zhiqiang Ruan^1,2^, Hui Yu^1,2^, Jieming Chen^1,2^, Mingjun Fan^1,2^, Yu Huang^1,2^, Min Wang^1,2^, Xiaomeng Zhao^1,2^, Guojun Hu^1,2^, Huanming Yang^2,13,14^, Jian Wang^2,13^, Jun Wang^2,13,15^, Xun Xu^2^, Linsheng Song^16^, Gangchun Xu^17^, Pao Xu^17^, Junmin Xu^2,18^, Stephen J. O’Brien^8,19^, László Orbán^5,20,21#^, Byrappa Venkatesh^4#^ & Qiong Shi^1,2,18#^

^1^Shenzhen Key Lab of Marine Genomics, Guangdong Provincial Key Lab of Molecular Breeding in Marine Economic Animals, Shenzhen 518083, China

^2^BGI-Shenzhen, Shenzhen 518083, China

^3^Key Laboratory of Tropical＆Subtropical Fishery Resource Application＆Cultivation, Ministry of Agriculture, Pearl River Fisheries Research Institute, Chinese Academy of Fishery Sciences, Guangzhou 510380, China

^4^Institute of Molecular and Cell Biology, A*STAR, Biopolis, Singapore 138673, Singapore

^5^ Reproductive Genomics Group, Temasek Life Sciences Laboratory, Singapore 117604, Singapore

^6^Laboratory of Chromosome Structure and Function, Department of Cytology and Histology, Biological Faculty, Saint Petersburg State University, Saint-Petersburg 198504, Russia

^7^Realbio Genomics Institute, Shanghai 200050, China

^8^Theodosius Dobzhansky Center for Genome Bioinformatics, Saint Petersburg State University, St. Petersburg 199004, Russia

^9^Institute of Molecular and Cellular Biology, Siberian Branch of the Russian Academy of Sciences, Novosibirsk 630090, Russia

^10^Department of Genomic Diversity and Evolution, Novosibirsk State University, Novosibirsk 630090, Russia

^11^Genomics Core Facility, Institute of Chemical Biology and Fundamental Medicine, Siberian Branch of the Russian Academy of Sciences, Novosibirsk 630090, Russia

^12^Environmental and Life Sciences Programme, Faculty of Science, Universiti Brunei Darussalam, BE1410 Brunei Darussalam

^13^James D. Watson Institute of Genome Science, Hangzhou 310008, China

^14^Princess Al Jawhara Center of Excellence in the Research of Hereditary Disorders, King Abdulaziz University, Jeddah, Saudi Arabia

^15^Department of Biology, University of Copenhagen, DK-2200 Copenhagen, Denmark

^16^Dalian Ocean University, Dalian 116023, China

^17^Freshwater Fisheries Research Center, Chinese Academy of Fishery Sciences, Wuxi 214081, China

^18^BGI-Zhenjiang Institute of Hydrobiology, Zhenjiang 212000, China

^19^Oceanographic Center, Nova Southeastern University Ft. Lauderdale, Ft Lauderdale, Florida 33004, USA

^20^Department of Animal Sciences and Breeding, Georgikon Faculty, University of Pannonia, H-8230 Keszthely, Hungary

^21^Centre for Comparative Genomics, Murdoch University, Murdoch, 6150 Australia

*These authors contributed equally to the work.

Correspondence should be addressed to shiqiong@genomics.cn, mcbbv@imcb.a-star.edu.sg and laszlo@tll.org.sg

**Supplementary Figures**


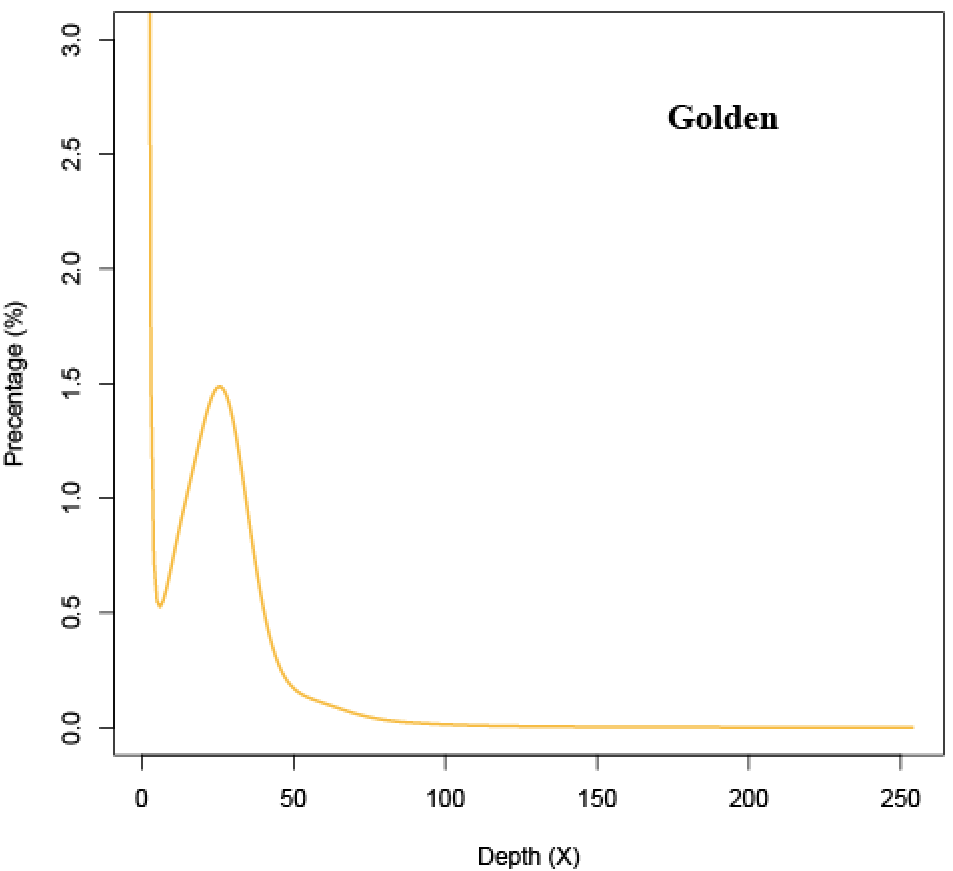

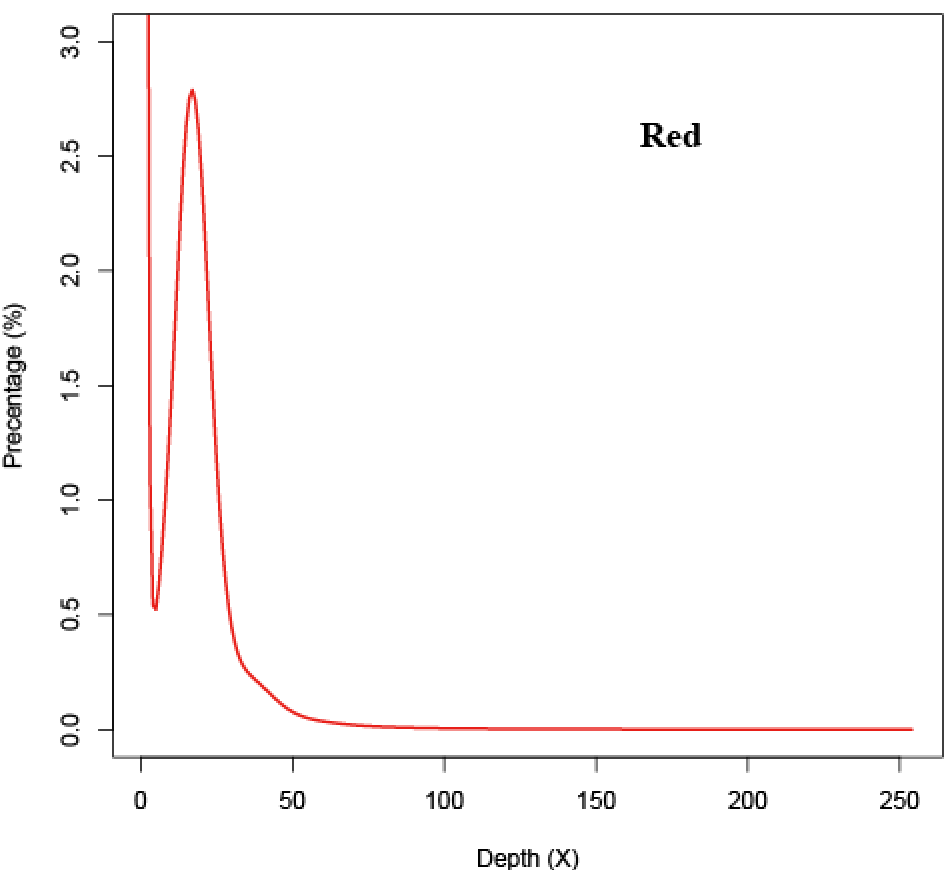


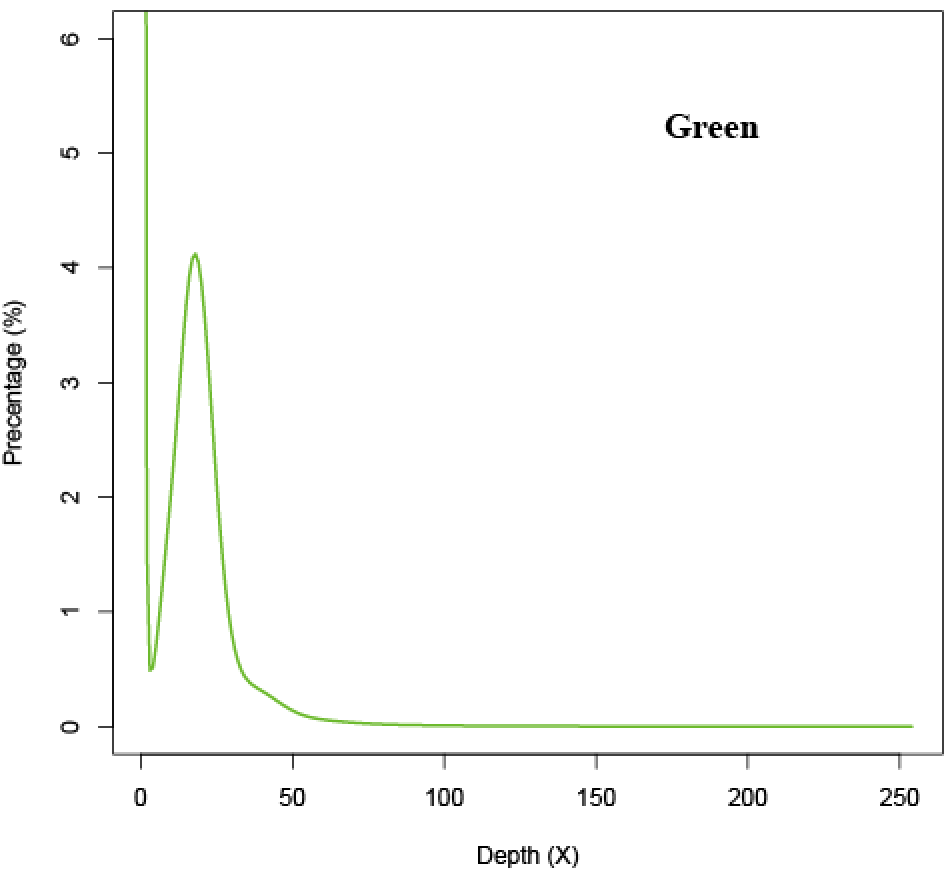


**Supplementary Figure 1.** **17-kmer analyses for prediction of genome sizes of the three Asian arowana varieties.** We estimated their genome sizes at 0.822 Gb (golden), 0.949 Gb (red) and 0.897 Gb (green), respectively.


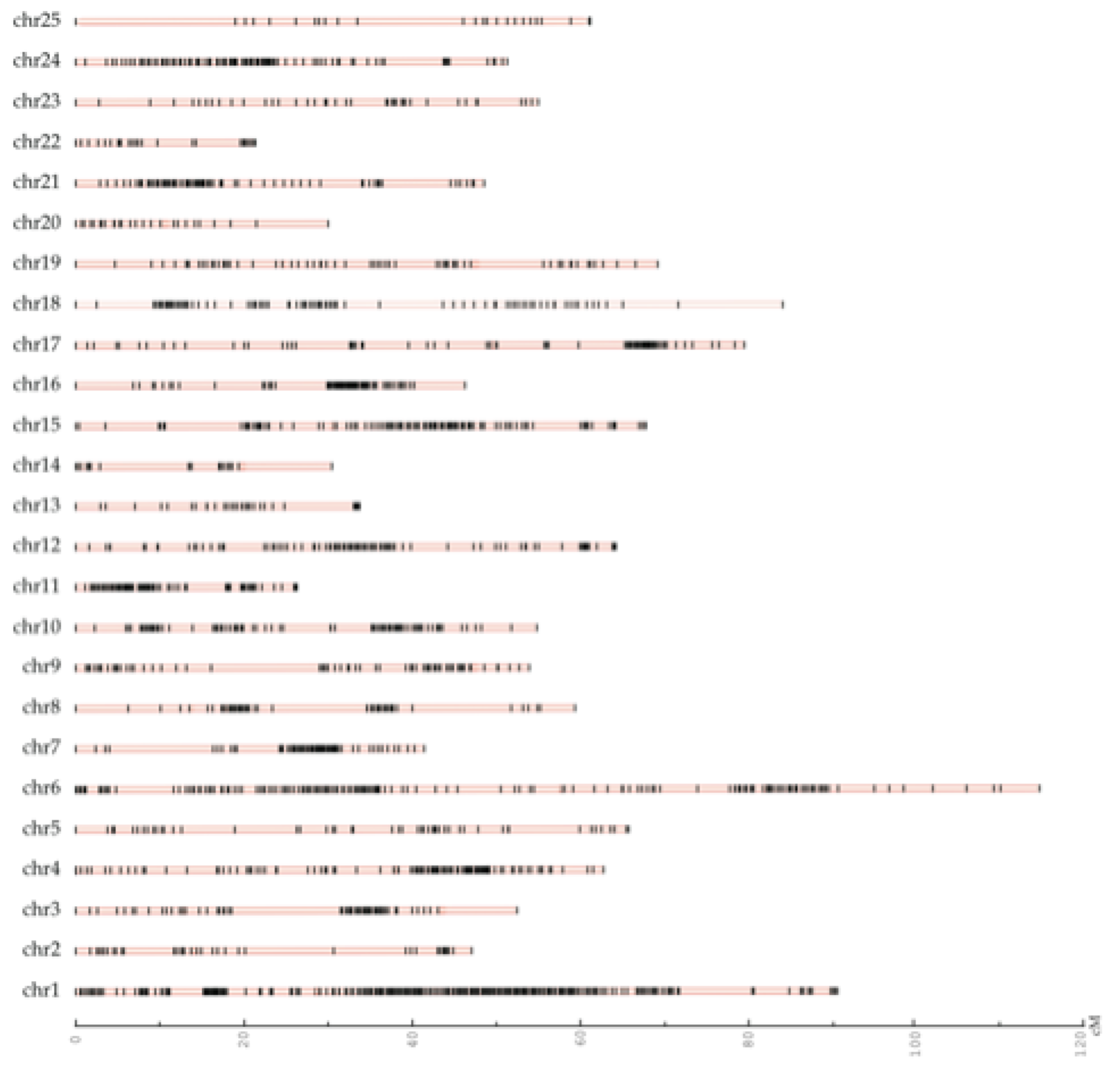


**Supplementary** Figure 2. **25 linkage groups (or pseudo-chromosomes) of golden arowana constructed by RAD sequencing and analysis.** The pink bars indicate the pseudo-chromosomes and the black lines represent the position of SNP markers in the linkage groups.


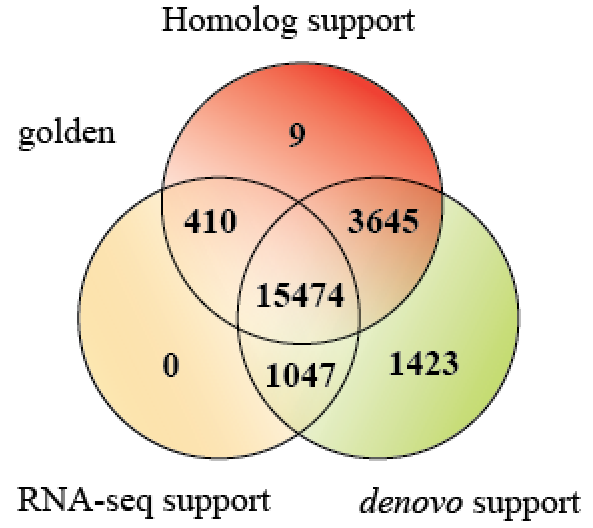

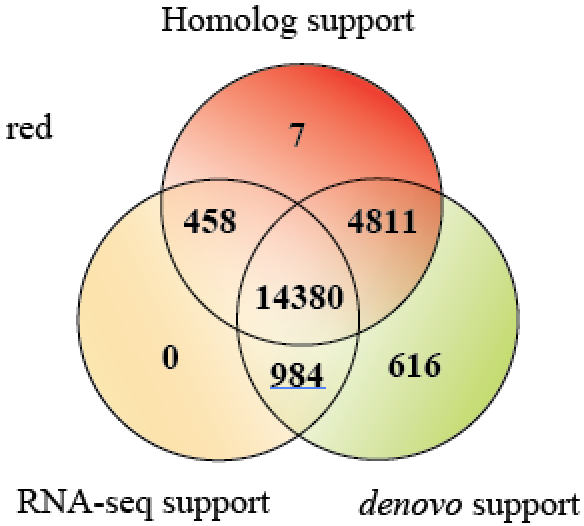


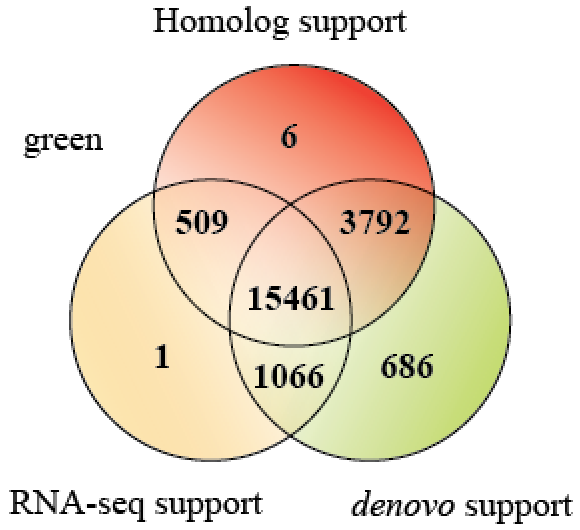


**Supplementary** Figure 3**. Gene models of the three arowana genomes are supported by three types of evidence.** Over 70% of the genes are supported by all the three types of evidence (homolog-based methods, RNA-seq and *de novo* prediction), confirming that the gene sets are highly reliable and elaborate.


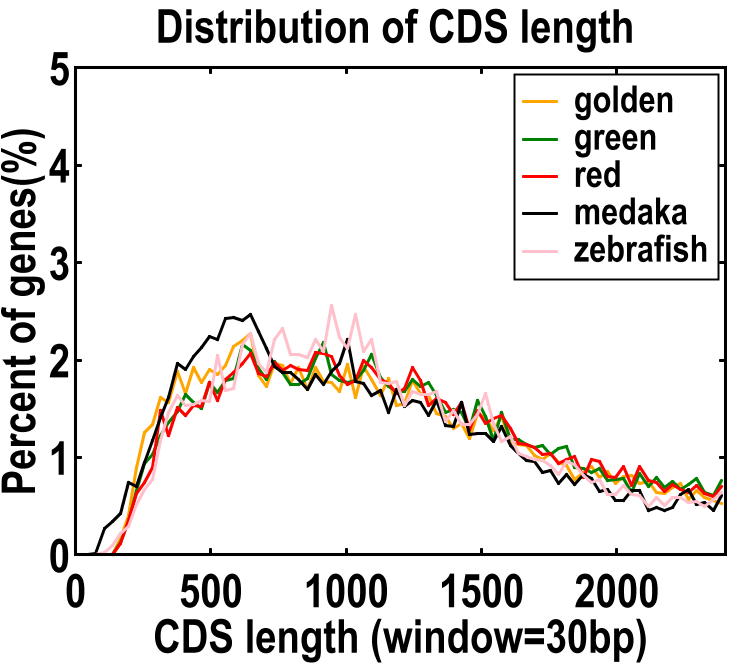

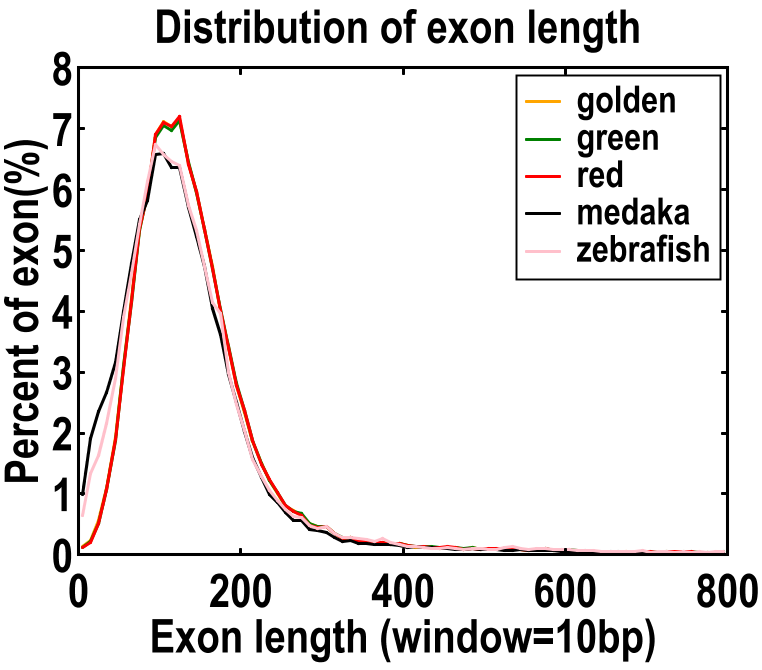


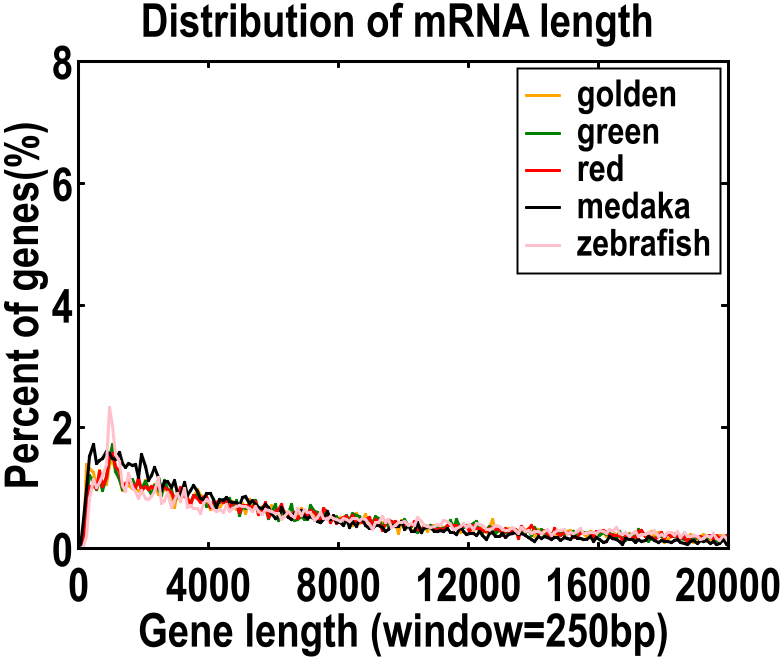

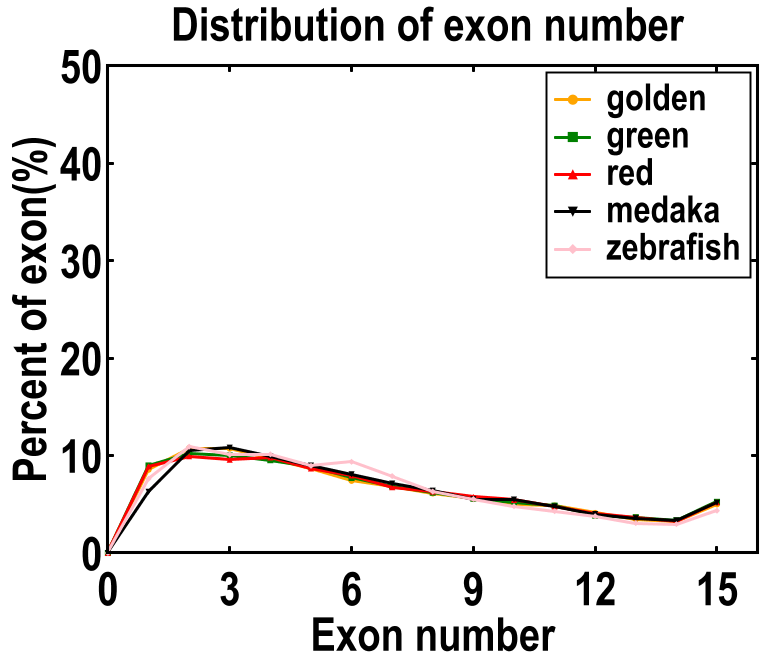


**Supplementary** Figure **4. Comparison of gene parameters among the three arowana varieties (golden, red and green), zebrafish and medaka.**

**
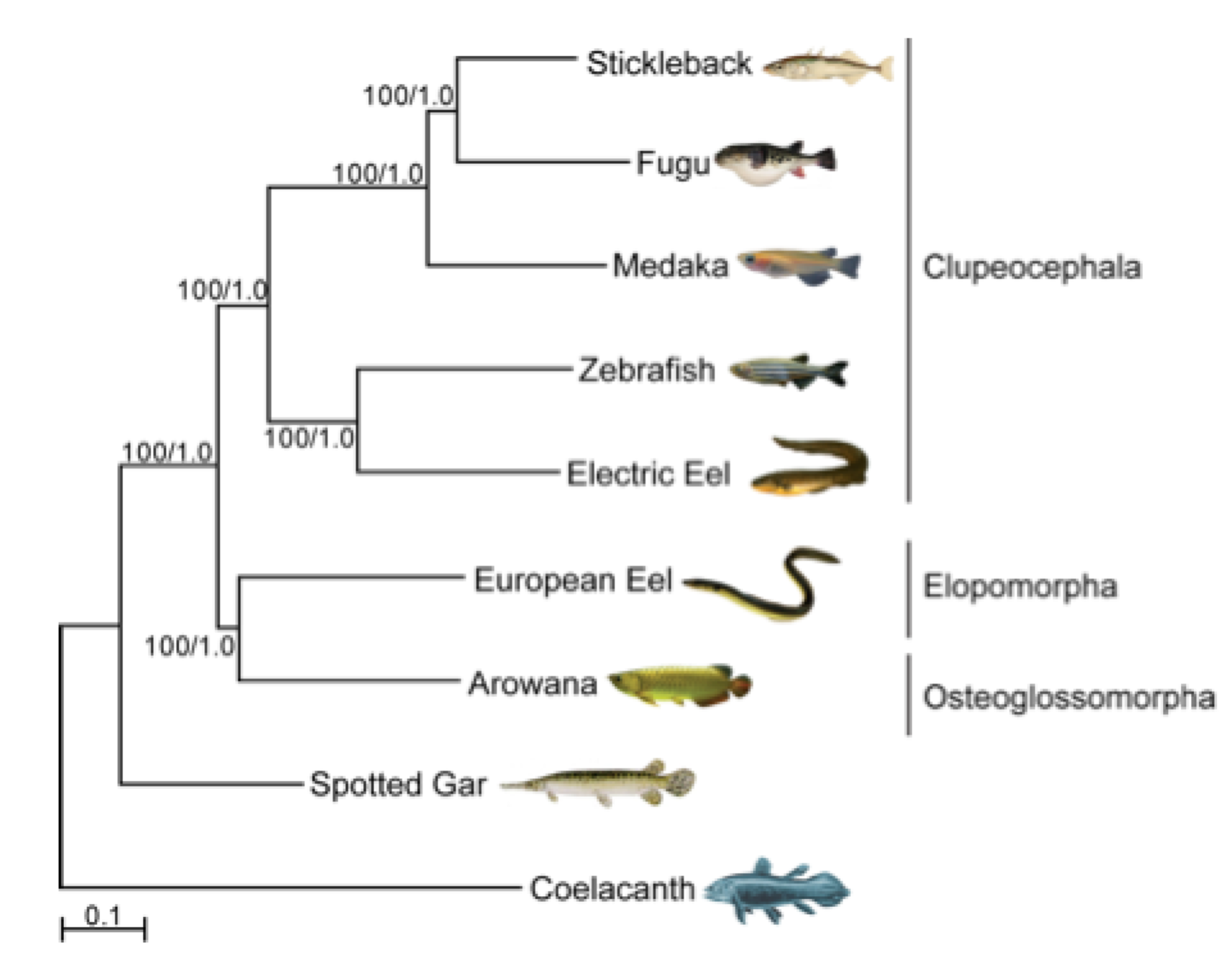
**

**Supplementary** Figure **5.** **Phylogenomic position of Asian arowana based on 2,463 one-to-one orthologues (2,473,368 nucleotide positions) from nine bony fish species.** Values shown at the nodes are Maximum Likelihood bootstrap percentages/Bayesian posterior probability values. The scale bar represents 0.1 substitutions per site.

**
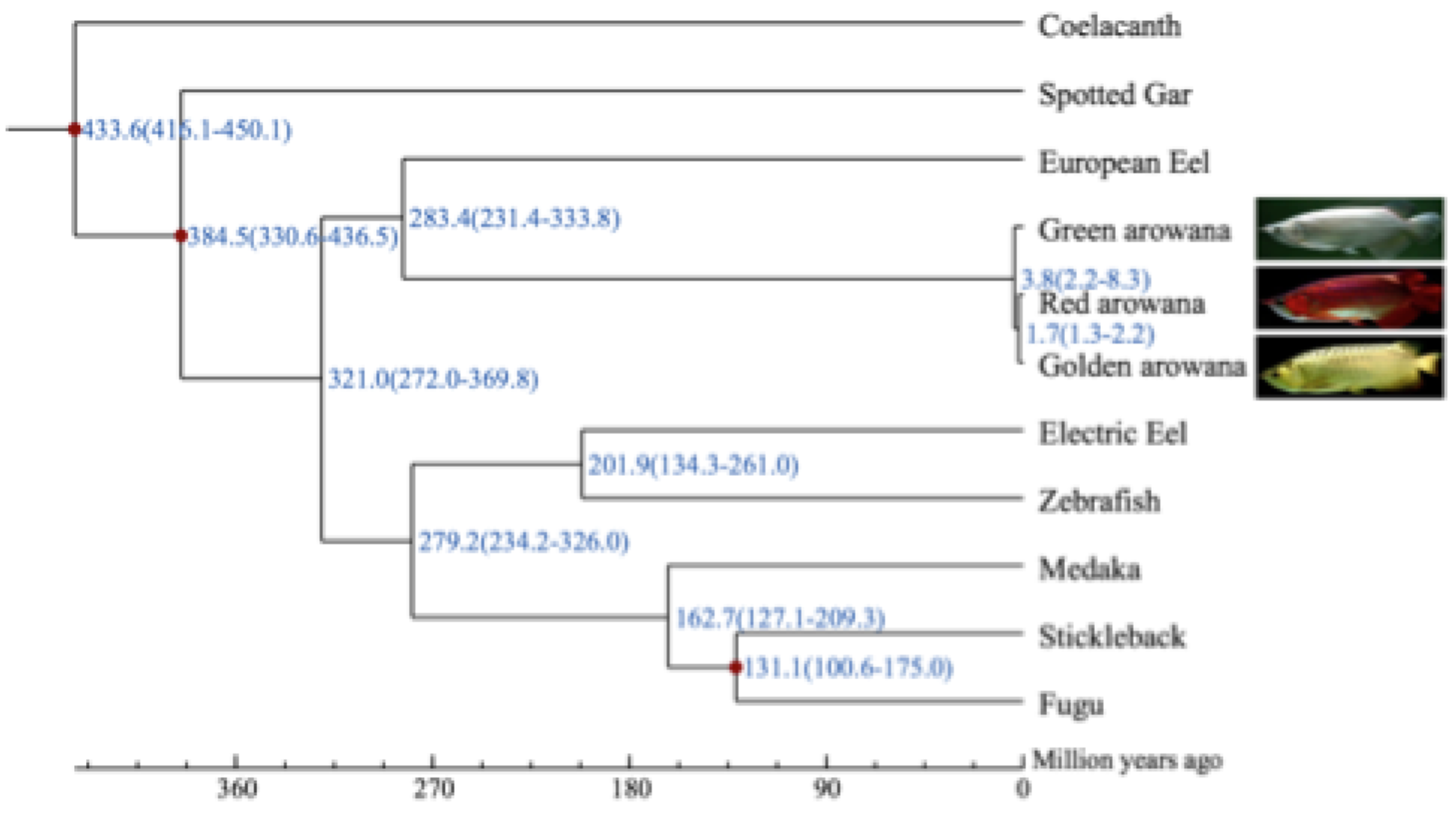
**

**Supplementary** Figure **6. The prediction of divergence time among nine species.** The blue numbers in branches are the divergence times (the unit is million years, Mya). Red dots in the three nodes indicate the fossil records that were used to be the calibration times (Tetraodontiform fossil from the Cenomanian[1], Tournasian Cosmoptychius as the earliest stem-group neopterygian[2], Probable divergence time between sarcopterygians and actinopterygians (450 MYA) based on both fossils and molecules[3], Psarolepis fossil (the earliest Sarcopterygii) from Ludlow Lophosteus and Andreolepis fossils from Ludlow[4])

**
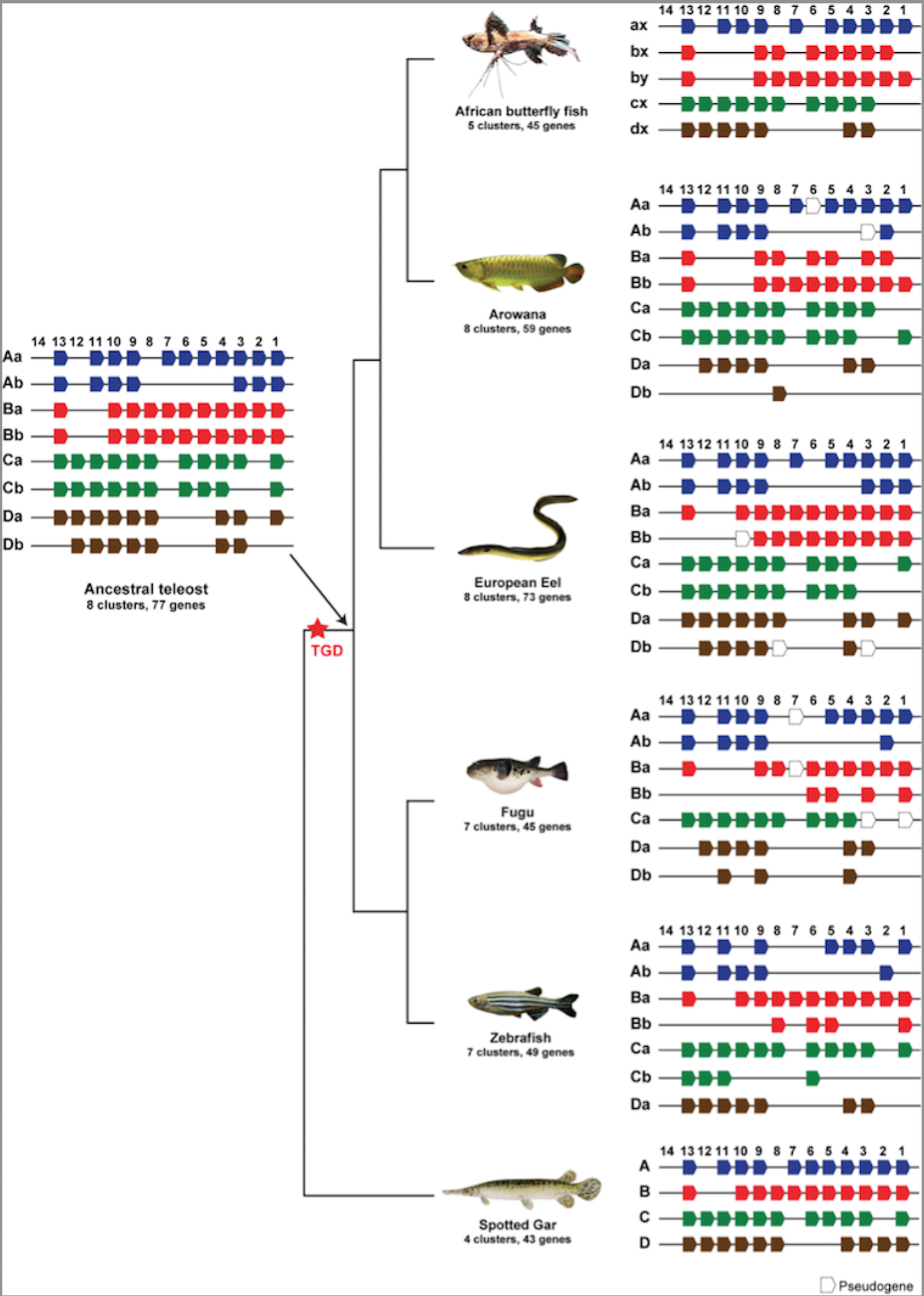
 Supplementary** Figure **7. Evolution of Hox gene clusters in teleost fishes.** Boxed arrows represent Hox genes with the direction of the arrows denoting the transcriptional orientation. The ancestral teleost fish Hox cluster (8 clusters containing 77 Hox genes) was reconstructed based on the Hox gene repertoire of five teleost fishes (African butterfly fish, Asian arowana, European eel, fugu and zebrafish).


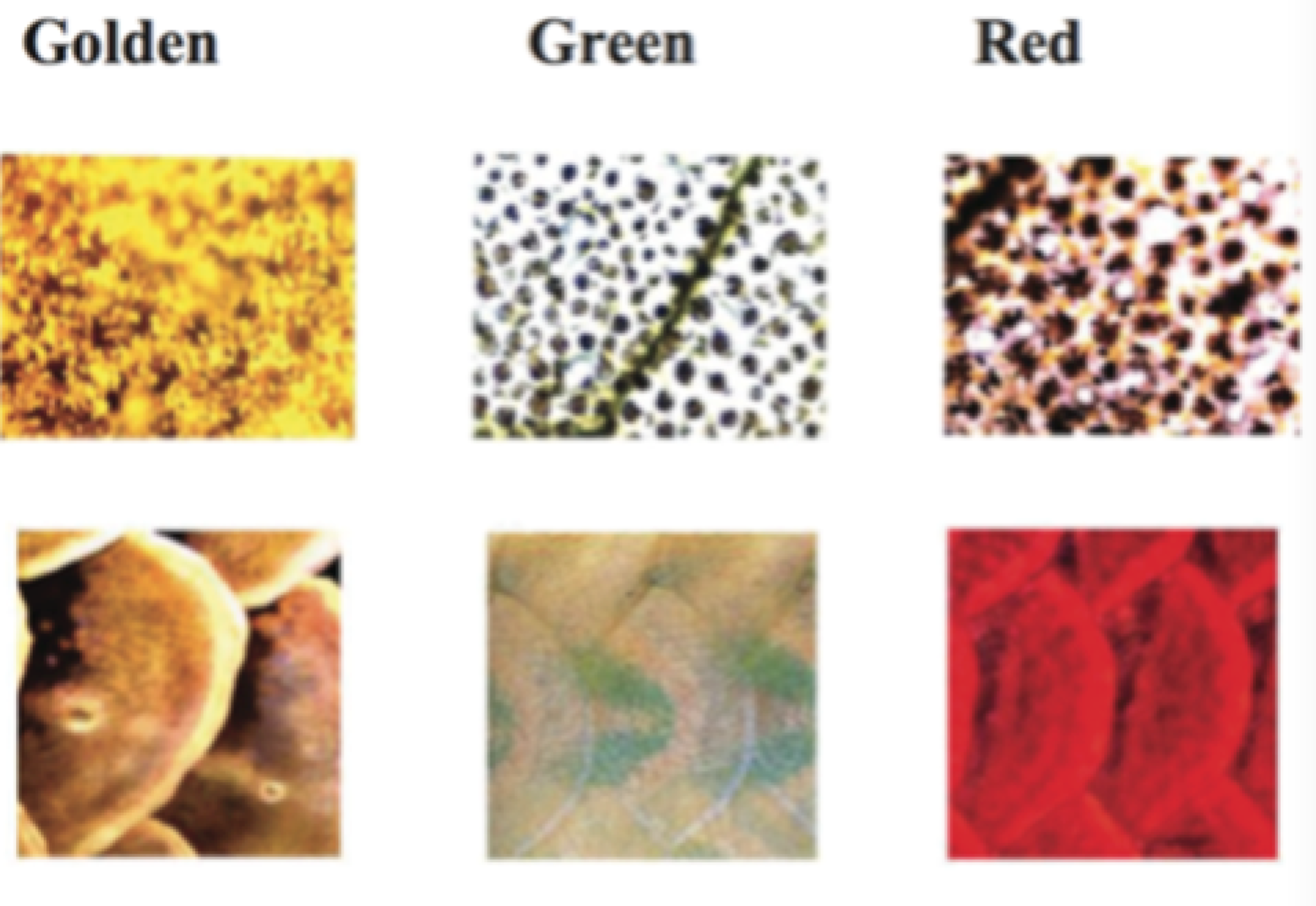


**Supplementary** Figure **8. The scales of each colour variety contain different chromatophores which strongly contribute to their unique phenotype.** Panels A-C: Scales of golden, green and red arowanas viewed under stereomicroscope (20X magnification, above row). Panels D-F: Close-up view of the scales using a DLR camera’s macro function (images from the Ph.D. Thesis of Alex Chang Kuok Wei [NUS, Singapore; 2010] with the permission of the author).


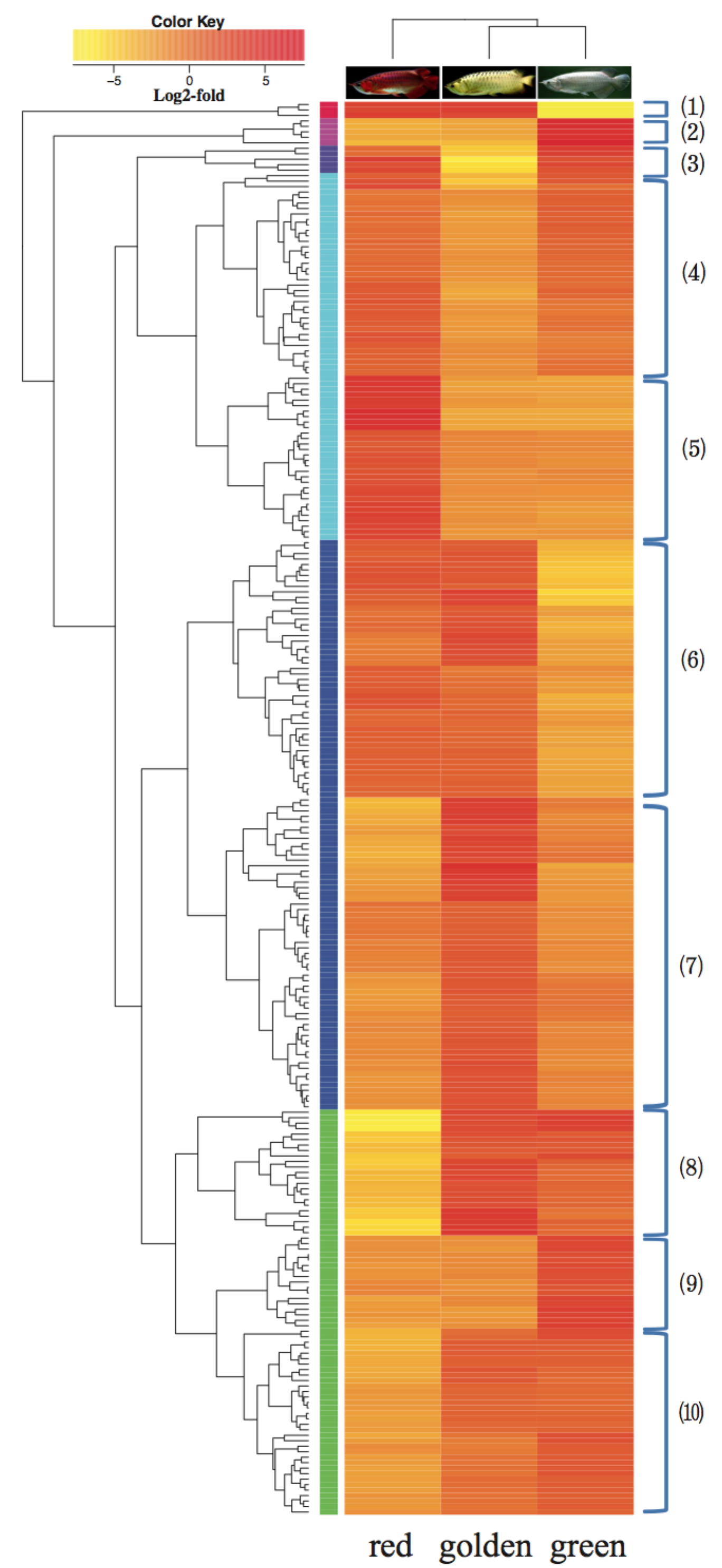


**Supplementary** Figure **9. Heatmap showing the expression trends and clustered groups of 260 genes differentially expressed in the scale and skin of the three varieties of Asian arowana.** This figure indicates that golden variety has more similar expression profiles to green one than red one. The threshold of significance test is 2-fold and *p*-value <0.05 performed by Trinity’s edgeR software[5].


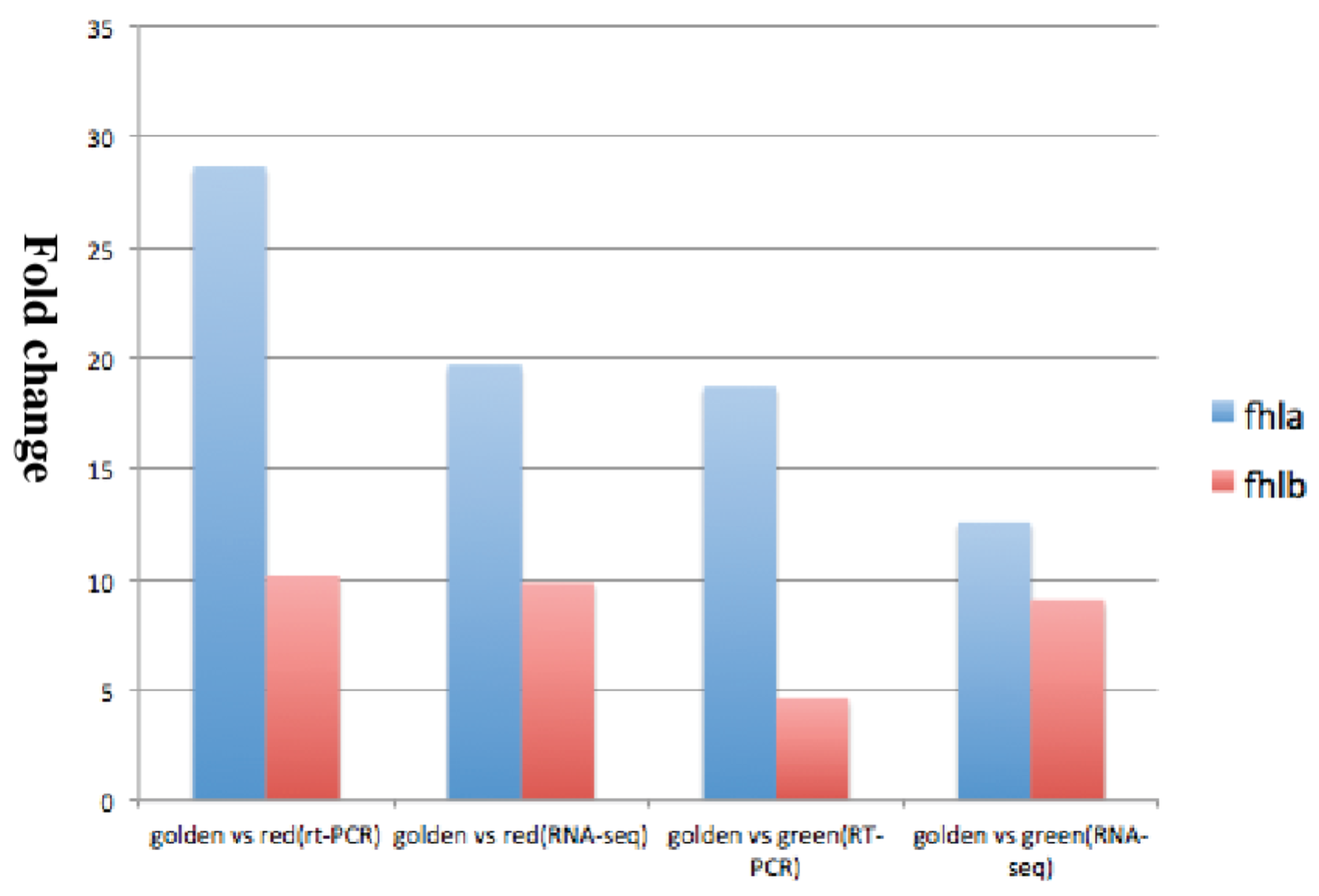


**Supplementary** Figure **10. The fold change of expression values of *fhla* and *fhlb* between golden and red varieties, as well as golden and green varieties based on RNASeq and validated by RT-PCR.** Both genes showed consistent expression patterns among the RT-PCR and RNA-seq.


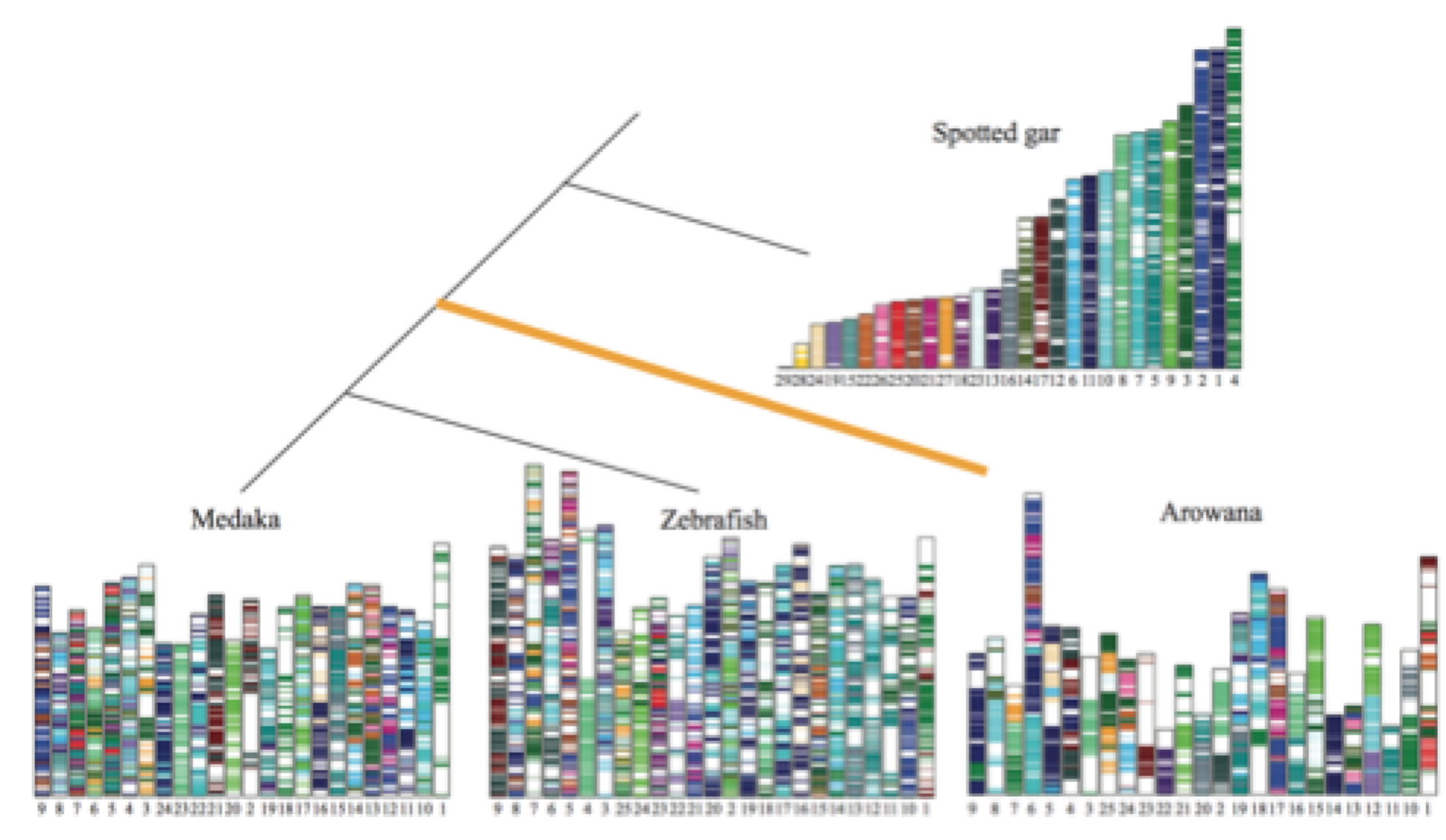


**Supplementary** Figure **11. Interchromosomal rearrangements in arowana, zebrafish and medaka chromosomes using the linkage groups (LGs) of spotted gar as reference.**


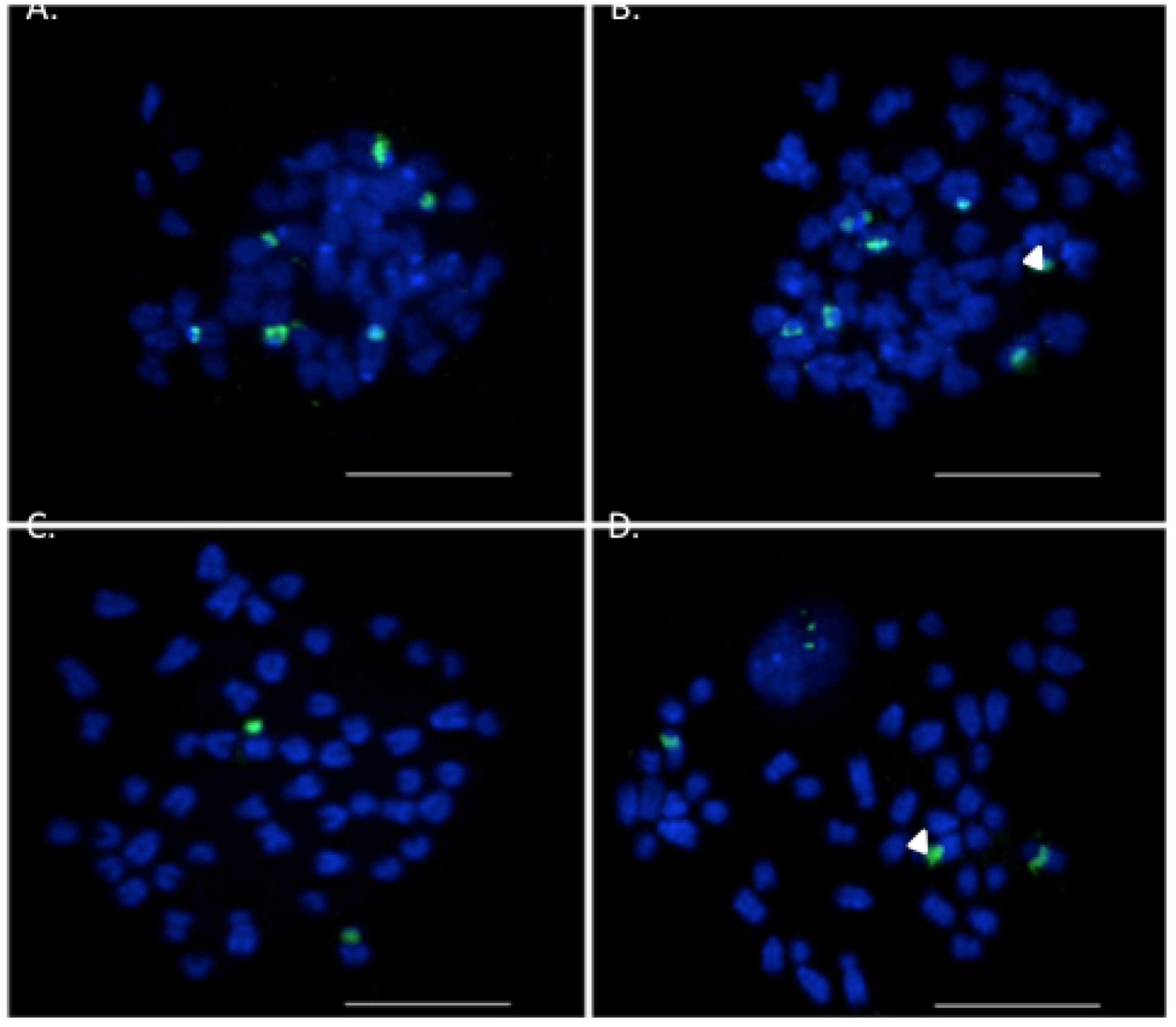


**Supplementary** Figure **12. Distribution of 5S rDNA and 18S rDNA visualized by fluorescence in situ hybridization (FISH) onto mitotic chromosomes of Asian arowana.** rDNA signals on metaphase spreads: (A) 5S rDNA – male; (B) 5S rDNA - female; (C) 18S rDNA – male; and (D) 18S rDNA - female. Chromosomes were contrasted by DAPI. White arrowheads indicate the putative W chromosome. Bar is 5 μm.FISH with the 5S rDNA probe showed signals in the centromeric or pericentromeric regions of three pairs of autosomes (A); In females, an additional signal was detected in the pericentromeric region of the large, female-specific chromosome mentioned above (B). For 18S rDNA, two signals were detected in males (C) and three signals, including the pericentromeric region of the putative W chromosome were found on female metaphase spreads (D).


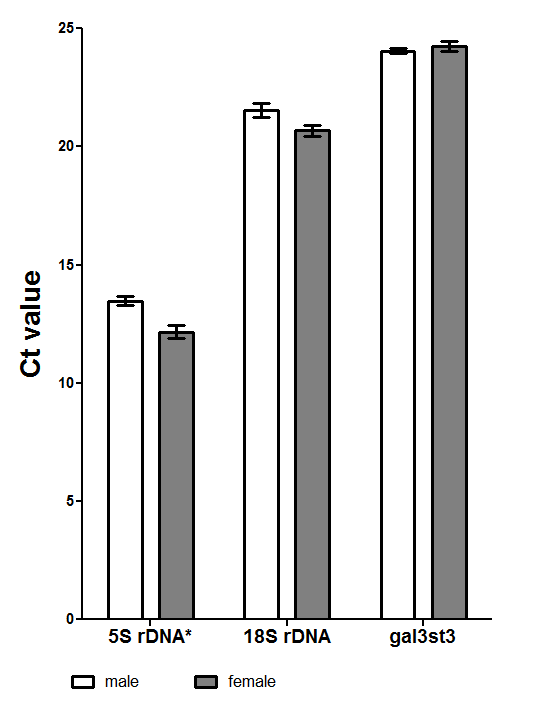


**Supplementary** Figure **13. qPCR-based quantification of 5S and 18S rDNA sequenences in the male and female Asian arowana genomes indicates that the size of the latter is larger than the former**. The Ct value for single copy genes is labeled by the red line. Each data point represents an average obtained from three qPCR reactions relative to the single-copy reference gene, *gal3st3 (galactose-3-O-sulfotransferase 3).* Asterisk indicates statistically significant difference between male and female (*p*>0.05; chi-test)

**
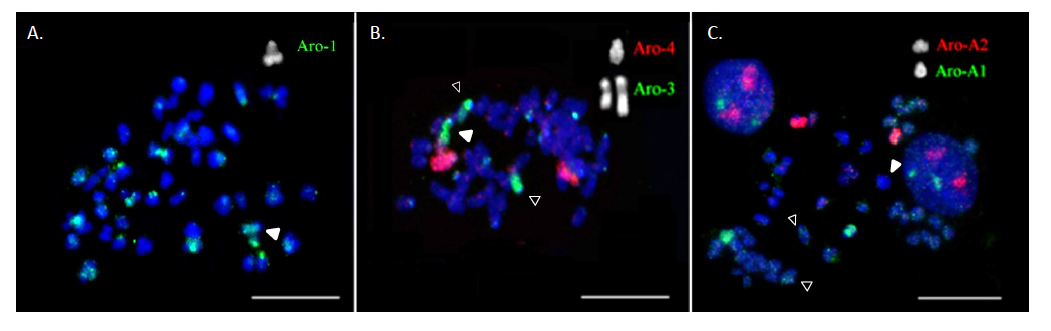
**

**Supplementary** Figure **14. Fluorescent in situ hybridization validates the origin of microdissected probes.** Five probes were generated from laser–microdissected chromosomal fragments: two from the large, putative female-specific chromosome (AroW1 & AroW3) and the remaining three from autosomes (AroA1, AroA2 & AroA4; controls). Panel A: AroW1 (green); Panel B: AroW3 (green) and AroA4 (red); Panel C: AroA1 (green) and AroA2 (red). Painted chromosomes stained by DAPI are shown in upper right corner of each panel. White arrowheads indicate the putative W-chromosome on all images. Transparent arrowheads label additional chromosomal pair(s) painted by the corresponding probes. Bar – 5 μm.

**Supplementary** Figure **15. Comparison between the 25 chromosomes of Asian arowana with the Z (panel A) and W (panel B) chromosome scaffolds of the tongue sole (*Cynoglossus semilaevis)*.** The comparisons were performed using Symap with default settings (minScore=30, minIdentity=70, tileSize=10, qMask=lower, maxIntron=10000). The Asian arowana chromosomes are labelled in black font, while the tongue sole chromosomes are labelled in blue font. Chr17 and Chr6 showed the largest aligned regions (89% and 53%) to tongue sole ChrZ, while Chr6 had the largest aligned region (40%) to tongue sole ChrW.


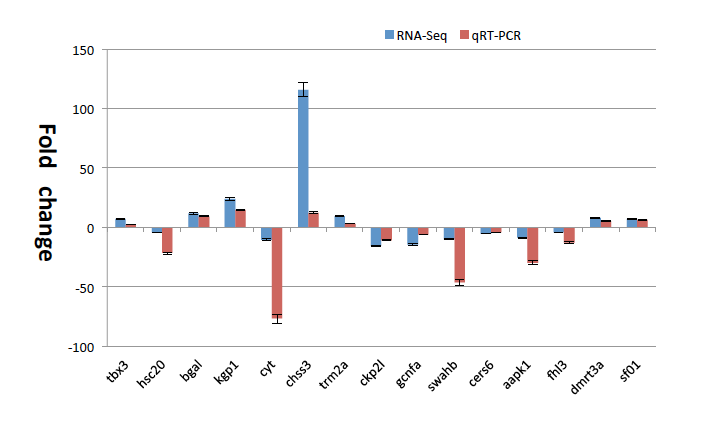


**Supplementary** Figure **16. Differential expressed genes between testis and ovary based on RNASeq and validated by qPCR.** A total of 15 DEGs was successfully validated by the qRT-PCR analysis. No genes showed inconsistent expression pattern with the transcriptome expression results. The Y-axis shows fold change of expression between testis and ovary (T/O).

**Supplementary Tables**

**Supplementary Table 1.** **Statistics of sequencing libraries and raw data from the genome of three arowanas varieties**

| Species | Insert Size | Total Data (Gb) | Read Length | Sequence coverage (X) |
| --- | --- | --- | --- | --- |
| Golden arowana | 170 bp | 24.7 | 100 | 30.1 |
|  | 500 bp | 20.3 | 100 | 24.7 |
|  | 800 bp | 13.8 | 100 | 16.8 |
|  | 2 kb | 22.5 | 49 | 27.4 |
|  | 5 kb | 10.6 | 49 | 12.9 |
|  | 10 kb | 9.9 | 49 | 12.0 |
|  | 20 kb | 6.6 | 49 | 8.0 |
|  | 40 kb | 4.7 | 49 | 5.7 |
| Total |  | 113.1 |  | 137.6 |
| Red arowana | 250 bp | 39.7 | 150 | 41.7 |
|  | 500 bp | 26.6 | 90 | 28.0 |
|  | 2 kb | 17.7 | 100 | 18.6 |
|  | 5 kb | 19.8 | 100 | 20.8 |
| Total |  | 103.8 |  | 109.6 |
| Green arowana | 250 bp | 32.7 | 150 | 36.3 |
|  | 500 bp | 27.6 | 90 | 30.7 |
|  | 2 kb | 15.8 | 100 | 17.6 |
|  | 5 kb | 14.4 | 100 | 16.0 |
| Total |  | 90.5 |  | 100.1 |

**Supplementary Table 2. Statistics of the filtered reads from the genome of three arowanas** varieties

| Species | Insert Size | Total Data (Gb) | Read Length | Sequence coverage (X) |
| --- | --- | --- | --- | --- |
| Golden arowana | 170 bp | 19.7 | 95 | 23.9 |
|  | 500 bp | 16.1 | 95 | 19.6 |
|  | 800 bp | 9.6 | 95 | 11.7 |
|  | 2 kb | 15.5 | 44 | 18.9 |
|  | 5 kb | 6.0 | 44 | 7.2 |
|  | 10 kb | 4.7 | 43 | 5.8 |
|  | 20 kb | 1.8 | 44 | 2.2 |
|  | 40 kb | 0.7 | 43 | 0.9 |
| Total |  | 74.1 |  | 90.1 |
| Red arowana | 250 bp | 31.7 | 144 | 33.3 |
|  | 500 bp | 20.4 | 85 | 21.5 |
|  | 2 kb | 7.7 | 94 | 8.11 |
|  | 5 kb | 15.8 | 94 | 16.6 |
| Total |  | 75.6 |  | 79.6 |
| Green arowana | 250 bp | 27.1 | 144 | 30.2 |
|  | 500 bp | 20.5 | 85 | 22.8 |
|  | 2 kb | 7.5 | 94 | 8.3 |
|  | 5 kb | 5.3 | 94 | 5.9 |
| Total |  | 60.4 |  | 67.1 |

**Supplementary Table 3. Statistics of the genome assemblies of three arowana varieties**

|  | **golden arowana** | | | **red arowana** | | **green arowana** | |
| --- | --- | --- | --- | --- | --- | --- | --- |
|  | **Scaffold (bp)** | **Contig (bp)** | **Scaffold (bp)** | | **Contig (bp)** | **Scaffold (bp)** | **Contig (bp)** |
| **N90** | 1,506,832 | 7,374 | 241,466 | | 14,666 | 275,265 | 14,427 |
| **N80** | 2,613,244 | 12,840 | 541,830 | | 25,427 | 591,242 | 25,911 |
| **N70** | 3,596,321 | 18,364 | 845,956 | | 35,795 | 949,766 | 37,393 |
| **N60** | 4,496,411 | 24,273 | 1,201,501 | | 47,390 | 1,435,603 | 49,818 |
| **N50** | 5,965,175 | 30,726 | 1,630,767 | | 60,192 | 1,852,627 | 62,797 |
| **N40** | 8,961,471 | 38,304 | 2,113,847 | | 75,011 | 2,329,365 | 78,910 |
| **N30** | 9,752,699 | 47,543 | 2,464,619 | | 93,016 | 3,061,240 | 97,916 |
| **N20** | 10,599,567 | 59,768 | 3,245,251 | | 116,576 | 3,911,715 | 123,535 |
| **N10** | 11,855,402 | 81,452 | 4,851,985 | | 159,024 | 5,092,394 | 169,694 |
| **Total length** | 779,258,895 | 741,391,717 | 752,855,112 | | 742,314,570 | 758,890,570 | 749,134,158 |
| **number>100** | 18,080 | 70,104 | 48,671 | | 72,055 | 74,063 | 96,660 |
| **number>2k** | 554 | 39,465 | 2,027 | | 21,545 | 1,804 | 20,838 |

**Supplementary Table 4. Assessing the completeness of gene regions in the three arowana genome assemblies by de novo assembled RNA-seq of skin tissue**

| golden skin | | | | | | | |
| --- | --- | --- | --- | --- | --- | --- | --- |
| Dataset | **Number** | **Total length** | **Covered by assembly (%)** | **with >90% sequence in one scaffold** | | **with >50% sequence in one scaffold** | |
|  |  |  |  | **Number** | **Percentage** | **Number** | **Percentage** |
| **>200bp** | 169,363 | 164,922,633 | 95.40 | 15,342 | 90.60 | 164,931 | 97.38 |
| **>500bp** | 89,682 | 140,089,723 | 95.10 | 80,719 | 90.01 | 86,707 | 96.68 |
| **>1000bp** | 53,652 | 114,374,236 | 94.81 | 47,724 | 88.95 | 51,601 | 96.17 |
| red skin | | | | | | | |
| Dataset | **Number** | **Total length** | **Covered by assembly (%)** | **with >90% sequence in one scaffold** | | **with >50% sequence in one scaffold** | |
|  |  |  |  | **Number** | **Percentage** | **Number** | **Percentage** |
| **>200bp** | 165,640 | 120,071,180 | 97.37 | 151,974 | 91.75 | 163,565 | 98.75 |
| **>500bp** | 70,609 | 91,534,661 | 97.25 | 65,171 | 92.30 | 69,688 | 98.70 |
| **>1000bp** | 35,440 | 66,668,595 | 96.99 | 32,394 | 91.40 | 34,900 | 98.48 |
| green skin | | | | | | | |
| Dataset | **Number** | **Total length** | **Covered by assembly (%)** | **with >90% sequence in one scaffold** | | **with >50% sequence in one scaffold** | |
|  |  |  |  | **Number** | **Percentage** | **Number** | **Percentage** |
| **>200bp** | 184943 | 17,0198,905 | 96.50 | 171,431 | 92.69 | 182,947 | 98.92 |
| **>500bp** | 91059 | 141,787,510 | 96.10 | 83,452 | 91.65 | 89,618 | 98.42 |
| **>1000bp** | 53915 | 115,302,119 | 95.61 | 48,529 | 90.01 | 52,784 | 97.90 |

**Supplementary Table 5. Statistics of** **Core Eukaryotic Genes (CEGs) evaluation of the genomes** of three arowana varieties

|  |  | **golden** | |  | | **red** | | | **green** | |
| --- | --- | --- | --- | --- | --- | --- | --- | --- | --- | --- |
|  | **Number** | | **Completeness (%)** | | **Number** | | **Completeness (%)** | **Number** | | **Completeness (%)** |
| **Total** | 244 | | 98.39 | | 246 | | 99.19 | 246 | | 99.19 |
| **Group1** | 66 | | 100.00 | | 65 | | 98.48 | 65 | | 98.48 |
| **Group2** | 55 | | 98.21 | | 56 | | 100.00 | 56 | | 100.00 |
| **Group3** | 60 | | 98.36 | | 60 | | 98.36 | 60 | | 98.36 |
| **Group4** | 63 | | 96.92 | | 65 | | 100.00 | 65 | | 100.00 |

**Supplementary Table 6. Overview of marker number, genetic distance and ph**ysical length of each pseudo chromosome.

| Pseudo-chromosome | Marker number | Genetic distance (cM) | Physical length (Mb) |
| --- | --- | --- | --- |
| Chr1 | 503 | 169.3 | 43.68 |
| Chr2 | 204 | 126.9 | 23.11 |
| Chr3 | 71 | 110.2 | 25.30 |
| Chr4 | 290 | 217.7 | 30.61 |
| Chr5 | 240 | 100.7 | 31.01 |
| Chr6 | 407 | 297.9 | 55.33 |
| Chr7 | 160 | 58.8 | 20.15 |
| Chr8 | 228 | 115.6 | 28.80 |
| Chr9 | 209 | 117.9 | 25.91 |
| Chr10 | 256 | 199.1 | 26.59 |
| Chr11 | 198 | 66.1 | 12.66 |
| Chr12 | 233 | 162.7 | 31.27 |
| Chr13 | 205 | 62.3 | 16.37 |
| Chr14 | 215 | 87.5 | 14.63 |
| Chr15 | 305 | 186.1 | 32.61 |
| Chr16 | 228 | 82.4 | 22.33 |
| Chr17 | 251 | 241.9 | 37.82 |
| Chr18 | 193 | 84.6 | 40.59 |
| Chr19 | 208 | 148.5 | 33.35 |
| Chr20 | 222 | 91.9 | 14.83 |
| Chr21 | 188 | 101.2 | 23.76 |
| Chr22 | 177 | 87.3 | 12.02 |
| Chr23 | 112 | 94.1 | 25.97 |
| Chr24 | 221 | 113.2 | 24.85 |
| Chr25 | 216 | 116.8 | 29.49 |
| Total | 5,617 | 3,240.7 | 683.04 |

**Supplementary Table 7. Statistics of predominant subfamilies of repetitive sequences in the genome of three arowana varieties**

| **Transposable element** | **golden** | | | **red** | | | | **green** | | | |
| --- | --- | --- | --- | --- | --- | --- | --- | --- | --- | --- | --- |
|  | **Number** | **#base (bp)** | **%G** | | **Number** | **#base (bp)** | **%G** | | **Number** | **#base (bp)** | **%G** |
| **DNA** |  |  |  | |  |  |  | |  |  |  |
| TcMar | 503528 | 65076617 | 8.35 | | 462405 | 59887091 | 7.95 | | 464266 | 59929709 | 7.90 |
| hAT | 58092 | 5661737 | 0.73 | | 48432 | 4183913 | 0.56 | | 59671 | 5629811 | 0.74 |
| Sola | 41312 | 4297656 | 0.55 | | 23765 | 2394681 | 0.32 | | 26068 | 2703026 | 0.36 |
| Novosib | 36822 | 4243379 | 0.54 | | 63327 | 7435182 | 0.99 | | 50812 | 6326973 | 0.83 |
| CMC | 41255 | 3821574 | 0.49 | | 65228 | 7113066 | 0.94 | | 81363 | 9114182 | 1.20 |
| Ginger | 26585 | 3198003 | 0.41 | | 19100 | 1961259 | 0.26 | | 17089 | 1789052 | 0.24 |
| **LINE** |  |  |  | |  |  |  | |  |  |  |
| L2 | 319395 | 42040665 | 5.39 | | 257565 | 34199576 | 4.54 | | 292912 | 39154929 | 5.16 |
| RTE | 153996 | 22252613 | 2.86 | | 172445 | 24775057 | 3.29 | | 172188 | 25348952 | 3.34 |
| Rex-Babar | 100900 | 15176409 | 1.95 | | 123131 | 18116478 | 2.41 | | 121469 | 17575172 | 2.32 |
| R2 | 15791 | 2783394 | 0.36 | | 17523 | 3103919 | 0.41 | | 19779 | 3348542 | 0.44 |
| R1 | 2108 | 158560 | 0.02 | | 10311 | 1255118 | 0.17 | | 4317 | 467154 | 0.06 |
| Other | 62830 | 11168694 | 1.43 | | 39184 | 7219606 | 0.96 | | 41005 | 8050799 | 1.06 |
| **LTR** |  |  |  | |  |  |  | |  |  |  |
| Gypsy | 209608 | 36404389 | 4.67 | | 239547 | 40536514 | 5.38 | | 216533 | 38257074 | 5.04 |
| Ngaro | 180209 | 27156593 | 3.48 | | 202839 | 30159771 | 4.01 | | 173496 | 23907999 | 3.15 |
| Other | 161763 | 30635498 | 3.93 | | 188370 | 34697209 | 4.61 | | 150575 | 27161015 | 3.58 |
| **SINE** |  |  |  | |  |  |  | |  |  |  |
| V | 382928 | 49351796 | 6.33 | | 358867 | 45947229 | 6.10 | | 349129 | 45703940 | 6.02 |
| L2 | 36948 | 2110181 | 0.27 | | 34560 | 1777349 | 0.24 | | 35680 | 1962188 | 0.26 |
| MIR | 10834 | 1529945 | 0.20 | | 11687 | 1690873 | 0.22 | | 18749 | 2611941 | 0.34 |
| Other | 12292 | 2146824 | 0.28 | | 15887 | 2650833 | 0.35 | | 19917 | 3250468 | 0.43 |
| **Satellite** | 1014 | 223565 | 0.029 | | 20070 | 2685333 | 0.36 | | 6891 | 986325 | 0.13 |
| **Simple_repeat** | 72875 | 9928389 | 1.27 | | 39158 | 5675577 | 0.75 | | 100152 | 13111011 | 1.73 |
| **Unclassified** | 9537 | 3671754 | 0.47 | | 6624 | 2085526 | 0.28 | | 8086 | 2430932 | 0.32 |
| **Total (Non-redundance)** | 2440622 | 213102045 | 27.34 | | 2420025 | 210237977 | 27.93 | | 2430147 | 212780616 | 28.04 |

**Supplementary Table 8. Gene annotation statistics of the genomes of three arowana varieties**

| **golden** | | **Number** | **Average Transcript Length (bp)** | **Average CDS Length (bp)** | **Average Exons per Gene** | **Average Exon Length (bp)** | **Average Intron Length (bp)** |
| --- | --- | --- | --- | --- | --- | --- | --- |
| ***De novo*** | AUGUSTUS | 26,835 | 13,294.20 | 1,383.94 | 8.10 | 170.79 | 1,676.79 |
|  | Genescan | 34,498 | 16,276.36 | 1,579.93 | 9.03 | 174.88 | 1,829.16 |
| **Homolog** | coelacanth | 30,370 | 6,831.41 | 1,136.74 | 5.87 | 193.65 | 1,169.33 |
|  | stickleback | 29,086 | 8,000.11 | 1,148.53 | 6.51 | 176.32 | 1,242.63 |
|  | human | 18,546 | 11,575.15 | 1,513.20 | 8.60 | 175.89 | 1,323.35 |
|  | zebrafish | 27,017 | 9,428.48 | 1,335.60 | 7.25 | 184.12 | 1,294.06 |
| medaka | | 34,352 | 6,707.48 | 1,037.53 | 5.63 | 184.13 | 1,223.37 |
| fugu | | 23,531 | 9,950.07 | 1,373.49 | 7.68 | 178.74 | 1,283.06 |
| half tongue | | 21,725 | 10,775.33 | 1,430.82 | 8.17 | 175.23 | 1,304.16 |
| greenpuffer | | 17,921 | 10,041.78 | 1,378.99 | 7.79 | 176.99 | 1,275.54 |
| **Transcriptome** |  | 37,962 | 13,363.68 | 2,716.52 | 8.87 | 306.41 | 1,353.62 |
| **GLEAN** | | 22,119 | 16,823.61 | 1,788.06 | 10.33 | 173.11 | 1,611.65 |
| **Final gene set** | | 22,016 | 16,861.75 | 1,794.26 | 10.37 | 173.08 | 1,608.66 |

| **red** | | **Number** | **Average Transcript Length (bp)** | **Average CDS Length (bp)** | **Average Exons per Gene** | **Average Exon Length (bp)** | **Average Intron Length (bp)** |
| --- | --- | --- | --- | --- | --- | --- | --- |
| ***De novo*** | AUGUSTUS | 27,659 | 12,463.21 | 1,374.72 | 7.85 | 175.20 | 1,619.54 |
|  | Genescan | 34,818 | 14,716.93 | 1,566.16 | 8.78 | 178.35 | 1,690.01 |
| **Homolog** | coelacanth | 26,503 | 7,222.64 | 1,210.46 | 6.34 | 190.88 | 1,125.56 |
|  | stickleback | 25,528 | 8,615.62 | 1,252.31 | 7.09 | 176.60 | 1,208.87 |
|  | human | 18,628 | 11,127.66 | 1,510.99 | 8.49 | 177.96 | 1,283.86 |
|  | zebrafish | 20,161 | 9,224.38 | 1,341.34 | 7.36 | 182.15 | 1,238.74 |
| medaka | | 28,090 | 7,449.70 | 1,152.65 | 6.31 | 182.62 | 1,185.51 |
| fugu | | 22,040 | 9,950.44 | 1,425.20 | 7.97 | 178.76 | 1,222.65 |
| half tongue | | 21,407 | 10,502.82 | 1,445.35 | 8.17 | 177.00 | 1,264.00 |
| greenpuffer | | 20,371 | 10,087.06 | 1,427.60 | 8.21 | 173.93 | 1,201.35 |
| **Transcriptome** |  | 35,631 | 12,437.60 | 2,560.09 | 8.91 | 287.30 | 1,248.59 |
| **GLEAN** | | 22,335 | 15,840.53 | 1,772.00 | 10.07 | 176.04 | 1,551.81 |
| **Final gene set** | | 21,256 | 16,183.93 | 1,839.83 | 10.45 | 176.00 | 1,517.31 |

| **green** | | **Number** | **Average Transcript Length (bp)** | **Average CDS Length (bp)** | **Average Exons per Gene** | **Average Exon Length (bp)** | **Average Intron Length (bp)** |
| --- | --- | --- | --- | --- | --- | --- | --- |
| ***De novo*** | AUGUSTUS | 27,810 | 12,464.81 | 1,372.95 | 7.82 | 175.61 | 1,626.77 |
|  | Genescan | 34,764 | 14,985.40 | 1,580.09 | 8.85 | 178.62 | 1,708.49 |
| **Homolog** | coelacanth | 26,116 | 7,344.85 | 1,213.68 | 6.36 | 190.88 | 1,144.26 |
|  | stickleback | 26,085 | 8,498.65 | 1,234.66 | 6.99 | 176.76 | 1,213.68 |
|  | human | 18,736 | 11,185.85 | 1,511.58 | 8.49 | 178.11 | 1,292.15 |
|  | zebrafish | 24,523 | 9,810.71 | 1,407.70 | 7.67 | 183.64 | 1,260.63 |
| medaka | | 28,664 | 7,463.86 | 1,145.70 | 6.25 | 183.27 | 1,203.13 |
| fugu | | 22,235 | 9,980.67 | 1,416.51 | 7.90 | 179.22 | 1,240.50 |
| half tongue | | 20,770 | 10,516.38 | 1,433.31 | 8.14 | 176.18 | 1,272.96 |
| greenpuffer | | 20,453 | 10,202.13 | 1,429.18 | 8.20 | 174.23 | 1,217.97 |
| **Transcriptome** |  | 37,674 | 13,842.57 | 3,153.81 | 9.28 | 339.75 | 1,290.49 |
| **GLEAN** | | 22,539 | 15,990.45 | 1,779.44 | 10.05 | 177.01 | 1,569.76 |
| **Final gene set** | | 21,524 | 16,299.18 | 1,842.96 | 10.41 | 176.99 | 1,535.81 |

**Supplementary Table 9. Skin expression value of each gene in the three arowana colour varieties**

**(see the separate Excel file)**

**Supplementary Table 10. Functional assignments of gene sets in the three genomes of three arowana varieties**

| **golden** | **Number** | **Percentage (%)** |
| --- | --- | --- |
| **Total** | 22,016 |  |
| **InterPro** | 18,313 | 83.18 |
| **GO** | 15,459 | 70.22 |
| **KEGG** | 15,645 | 71.06 |
| **Swissprot** | 20,065 | 91.14 |
| **TrEMBL** | 20,509 | 93.15 |
| **Annotated** | 21,629 | 98.24 |
| **Unanotated** | 387 | 1.76 |

| **red** | **Number** | **Percentage (%)** |
| --- | --- | --- |
| **Total** | 21,256 |  |
| **InterPro** | 18,417 | 86.64 |
| **GO** | 15,522 | 73.02 |
| **KEGG** | 16,834 | 79.20 |
| **Swissprot** | 20,188 | 94.98 |
| **TrEMBL** | 20,640 | 97.10 |
| **Annotated** | 20,679 | 97.29 |
| **Unanotated** | 577 | 2.71 |

| **green** | **Number** | **Percentage (%)** |
| --- | --- | --- |
| **Total** | 21,524 |  |
| **InterPro** | 18,328 | 85.15 |
| **GO** | 15,474 | 71.89 |
| **KEGG** | 16,903 | 78.53 |
| **Swissprot** | 20,284 | 94.24 |
| **TrEMBL** | 20,733 | 96.33 |
| **Annotated** | 20,762 | 96.46 |
| **Unanotated** | 762 | 3.54 |

**Supplementary Table 11. Evaluation of alternate topologies for the 9-species dataset using CONSEL**

| Rank | Topology | AU | NP | BP | PP | KH | SH | wKH | wSH |
| --- | --- | --- | --- | --- | --- | --- | --- | --- | --- |
| 1 | (C,(E,O)) | 1.000 | 1.000 | 1.000 | 1.000 | 1.000 | 1.000 | 1.000 | 1.000 |
| 2 | (O,(C,E)) | 3e-05 | 6e-06 | 0 | 0 | 0 | 0 | 0 | 0 |
| 3 | (E,(C,O)) | 5e-47 | 1e-16 | 0 | 0 | 0 | 0 | 0 | 0 |
| C - Clupeocephala (fugu, stickleback, medaka, zebrafish, electric eel); E - Elopomorpha (European eel); O - Osteoglossomorpha (arowana) | | | | | | | | | |

Three selected topologies were evaluated using several tests as implemented in the program CONSEL. A concatenated nucleotide alignment of 2463 one-to-one core orthologs was used for topology testing. The tests used are: approximately unbiased test (AU), bootstrap probability (NP, BP), Bayesian posterior probability (PP), Kishino-Hasegawa test (KH), Shimodaira-Hasegawa test (SH), weighted KH test (wKH) and weighted SH test (wSH). p-values derived from these tests for the various topologies are shown (higher is better). All the tests ranked “Elopomorpha sister to Osteoglossomorpha” as the most likely topology. This congruence in the top-ranked topology between different tests is a strong support for this topology. This topology was also inferred by phylogenetic analysis using Maximum likelihood and Bayesian inference methods (see Additional File2: Figure S5).

**Supplementary Table 12. Evaluation of alternate topologies for the 12-species dataset using CONSEL**

| Rank | Topology | AU | NP | BP | PP | KH | SH | wKH | wSH |
| --- | --- | --- | --- | --- | --- | --- | --- | --- | --- |
| 1  2  3 | (C,(E,O))  (E,(C,O))  (O,(C,E)) | 1.000  5e-04  0.002 | 0.999  0.001  4e-06 | 0.999  0.001  0 | 1.000  3e-71  3e-160 | 0.999  0.001  0 | 1.000  0.001  0 | 0.999  0.001  0 | 1.000  0.002  0 |
| C, Clupeocephala (fugu, stickleback, medaka, zebrafish, electric eel); E, Elopomorpha (European eel, tarpon); O - Osteoglossomorpha (arowana, butterflyfish, knifefish) | | | | | | | | | |

Three selected topologies were evaluated using several tests as implemented in the program CONSEL. A concatenated nucleotide alignment of 418 one-to-one core orthologs was used for topology testing. The tests used are: approximately unbiased test (AU), bootstrap probability (NP, BP), Bayesian posterior probability (PP), Kishino-Hasegawa test (KH), Shimodaira-Hasegawa test (SH), weighted KH test (wKH) and weighted SH test (wSH). p-values derived from these tests for the various topologies are shown (higher is better). All the tests ranked “Elopomorpha sister to Osteoglossomorpha” as the most likely topology. This congruence in the top-ranked topology between different tests is a strong support for this topology. This topology was also inferred by phylogenetic analysis using Maximum likelihood and Bayesian inference methods (see Figure 3).

**Supplementary Table 13. Gene synteny around the teleost fish Hox clusters.** The arowana Hox clusters were assigned to the Clupeocephalan paralog clusters ‘a’ or ‘b’ based on unique patterns of syntenic genes flanking the Hox clusters in arowana and representative teleost fishes. Genes informative in terms of assignment to the ‘a’ or ‘b’ paralog clusters are shaded.

| HoxAa locus | Arowana_scaf34 |  |  | Tax1bp1a | Hibadha | Evx1 | **HoxAa** |  | Snx10a | Cbx3a | Nfe2l |  |  |  |  |  |
| --- | --- | --- | --- | --- | --- | --- | --- | --- | --- | --- | --- | --- | --- | --- | --- | --- |
|  | Zebrafish |  |  | Tax1bp1a | Hibadha | Evx1 | **HoxAa** |  |  |  |  |  |  |  |  |  |
|  | Medaka |  |  | Tax1bp1a | Hibadha | Evx1 | **HoxAa** |  |  | Cbx3a | Nfe2l |  |  |  |  |  |
|  | Fugu |  |  | Tax1bp1a | Hibadha | Evx1 | **HoxAa** |  | Snx10a | Cbx3a | Nfe2l |  |  |  |  |  |
|  | Stickleback |  |  | Tax1bp1a | Hibadha | Evx1 | **HoxAa** |  | Snx10a | Cbx3a | Nfe2l |  |  |  |  |  |
|  | European eel |  |  |  |  | Evx1 | **HoxAa** |  |  |  |  |  |  |  |  |  |
|  |  |  |  |  |  |  |  |  |  |  |  |  |  |  |  |  |
| HoxAb locus | Arowana_scaf175 |  |  | Tax1bp1b | Hibadhb |  | **HoxAb** | Skap2 | Snx10b | Cbx3b |  | Pde11 |  |  |  |  |
|  | Zebrafish |  |  | Tax1bp1b | Hibadhb |  | **HoxAb** | Skap2 | Snx10b | Cbx3b |  | Pde11 |  |  |  |  |
|  | Medaka |  |  | Tax1bp1b | Hibadhb |  | **HoxAb** | Skap2 | Snx10b | Cbx3b |  | Pde11 |  |  |  |  |
|  | Fugu |  |  | Tax1bp1b | Hibadhb |  | **HoxAb** | Skap2 | Snx10b | Cbx3b |  |  |  |  |  |  |
|  | Stickleback |  |  | Tax1bp1b | Hibadhb |  | **HoxAb** | Skap2 | Snx10b | Cbx3b |  | Pde11 |  |  |  |  |
|  | European eel |  |  | Tax1bp1b | Hibadhb |  | **HoxAb** | Skap2 | Snx10b | Cbx3b |  | Pde11 |  |  |  |  |
|  |  |  |  |  |  |  |  |  |  |  |  |  |  |  |  |  |
| HoxBa locus | Arowana_scaf108 |  |  |  | Atp5g | Eve1 | **HoxBa** |  |  | Cbx1a |  | Osbpl6 | Znf |  |  |  |
|  | Zebrafish |  | Ube2za |  | Atp5g | Eve1 | **HoxBa** |  |  | Cbx1a | Nfe2la |  |  |  |  |  |
|  | Medaka |  | Ube2za |  |  | Eve1 | **HoxBa** |  |  | Cbx1a | Nfe2la | Praf2 |  |  |  |  |
|  | Fugu |  | Ube2za |  | Atp5g | Eve1 | **HoxBa** |  |  |  | Nfe2la |  |  |  |  |  |
|  | Stickleback |  | Ube2za |  | Atp5g |  | **HoxBa** |  |  | Cbx1a | Nfe2la | Praf2 |  |  |  |  |
|  | European eel |  | Ube2za |  | Atp5g | Eve1 | **HoxBa** |  |  |  |  |  |  |  |  |  |
|  |  |  |  |  |  |  |  |  |  |  |  |  |  |  |  |  |
| HoxBb locus | Arowana_scaf84 | Snf8 | Ube2zb | Tax1bp1 |  |  | **HoxBb** | Skap1 | Snx11 |  | Nfe2lb |  |  |  |  |  |
|  | Zebrafish |  |  | Srcap |  | Ttll6 | **HoxBb** | Skap1 |  |  |  |  |  |  |  |  |
|  | Medaka | Snf8 |  |  | Calcoco2 | Ttll6 | **HoxBb** | Skap1 | Snx11 |  |  |  |  |  |  |  |
|  | Fugu |  |  |  | Calcoco2 | Ttll6 | **HoxBb** | Skap1 |  |  |  |  |  |  |  |  |
|  | Stickleback |  |  |  | Calcoco2 | Ttll6 | **HoxBb** | Skap1 | Snx11 | Cbx1b | Nfe2lb |  |  |  |  |  |
|  | European eel |  |  |  |  | Ttll6 | **HoxBb** | Skap1 | Snx11 |  |  |  |  |  |  |  |
|  |  |  |  |  |  |  |  |  |  |  |  |  |  |  |  |  |
| HoxCa locus | Arowana_scaf66 |  | Mfsd5 | Nr1d4a | Rarga | Calcoco1a | **HoxCa** |  | Cbx5a | Hnrnpa1b | Nfe2 |  | Znf740b | Csada | Lima1a |  |
|  | Zebrafish |  | Mfsd5 | Nr1d4a | Rarga | Calcoco1a | **HoxCa** |  | Cbx5a | Hnrnpa1b | Nfe2 | Copz1 | Znf740b | Csada | Lima1a |  |
|  | Medaka |  | Mfsd5 | Nr1d4a | Rarga | Calcoco1a | **HoxCa** |  | Cbx5a | Hnrnpa1b |  | Copz1 | Znf740b | Csada |  |  |
|  | Fugu |  | Mfsd5 | Nr1d4a | Rarga | Calcoco1a | **HoxCa** |  | Cbx5a | Hnrnpa1b | Nfe2 | Copz1 | Znf740b | Csada |  |  |
|  | Stickleback |  | Mfsd5 | Nr1d4a | Rarga | Calcoco1a | **HoxCa** |  | Cbx5a | Hnrnpa1b | Nfe2 | Copz1 |  |  |  |  |
|  | European eel |  |  |  | Rarga | Calcoco1a | **HoxCa** | Smug1 | Cbx5a | Hnrnpa1b |  | Copz1 | Znf740b |  |  |  |
|  |  |  |  |  |  |  |  |  |  |  |  |  |  |  |  |  |
| HoxCb locus | Arowana_scaf83 |  |  | Nr1d4b | Rargb | Calcoco1b | **HoxCb** | Smug1 | Cbx5b | Hnrnpa1a |  |  | Znf740a | Csadb | Lima1b | Spryd3 |
|  | Zebrafish |  |  | Nr1d4b | Rargb | Calcoco1b | **HoxCb** | Smug1 |  | Hnrnpa1a |  |  | Znf740a |  | Lima1b |  |
|  | Medaka |  |  | Nr1d4b | Rargb | Calcoco1b |  | Smug1 |  |  |  |  | Znf740a |  | Lima1b | Spryd3 |
|  | Fugu | Slc4a8 | Scn8a |  | Rargb | Calcoco1b |  | Smug1 |  |  |  |  | Znf740a |  | Lima1b | Spryd3 |
|  | Stickleback |  |  | Nr1d4b | Rargb | Calcoco1b |  | Smug1 |  |  |  |  | Znf740a |  | Lima1b | Spryd3 |
|  | European eel |  |  |  | Rargb |  | **HoxCb** |  |  |  |  |  |  |  |  |  |
|  |  |  |  |  |  |  |  |  |  |  |  |  |  |  |  |  |
| HoxDa locus | Arowana_scaf95 | Chn1 | Atf2a | Atp5g3a | Lnpa | Evx2 | **HoxD** |  | Hnrnpa3 | Nfe2l2a | Agps | Pde11a |  |  |  |  |
|  | Zebrafish | Chn1 | Atf2a | Atp5g3a | Lnpa | Evx2 | **HoxDa** |  | Hnrnpa3 | Nfe2l2a | Agps | Pde11a |  |  |  |  |
|  | Medaka | Chn1 | Atf2a | Atp5g3a | Lnpa | Evx2 | **HoxDa** |  | Hnrnpa3 | Nfe2l2a | Agps | Pde11a |  |  |  |  |
|  | Fugu | Chn1 | Atf2a | Atp5g3a | Lnpa | Evx2 | **HoxDa** |  | Hnrnpa3 | Nfe2l2a | Agps | Pde11a |  |  |  |  |
|  | Stickleback | Chn1 | Atf2a | Atp5g3a | Lnpa | Evx2 | **HoxDa** |  | Hnrnpa3 | Nfe2l2a | Agps | Pde11a |  |  |  |  |
|  | European eel |  |  |  |  | Evx2 | **HoxDa** | Mtx2 | Hnrnpa | Nfe2l2a | Agps |  |  |  |  |  |
|  |  |  |  |  |  |  |  |  |  |  |  |  |  |  |  |  |
| HoxDb locus | Arowana_scaf102 |  | Atf2b | Atp5g3b | Lnpb |  | **HoxD** | Mtx2 |  |  | Agps |  |  |  |  |  |
|  | Zebrafish | Chrna1 |  | Atp5g3b | Lnpb |  |  | Mtx2 |  | Nfe2l2b |  |  |  |  |  |  |
|  | Medaka | Chrna1 |  |  | Lnpb |  | **HoxDb** | Mtx2 |  |  |  |  |  |  |  | Gpr155b |
|  | Fugu | Chrna1 |  |  | Lnpb |  | **HoxDb** | Mtx2 |  |  |  |  | Cdh5 | Bean1 | Tmem41a | Gpr155b |
|  | Stickleback | Chrna1 |  |  | Lnpb |  | **HoxDb** | Mtx2 |  |  |  |  | Cdh5 | Bean1 | Tmem41ab | Gpr155b |
|  | European eel | Chrna1 | Atf2b | Atp5g3b | Lnpb |  | **HoxDb** |  | Cycsb |  |  |  |  |  |  |  |

**Supplementary Table 14. Differentially expressed genes and their corresponding function in skin and scale tissues of the three arowanas colour varieties**

(see separate Excel file)

**Supplementary Table 15. Double-Conserved Syntenies (DCSs) between the human and golden arowana genomes.** Each row shows the values associated with one DCS. The columns display, from left to right, the human chromosome number, chromosome numbers of arowana duplicate chromosomes, numbers of orthologous genes on the two arowana duplicate chromosomes in the DCS.

| Human chromosome | arowana a chromosome | arowana b chromosome | a orthologues | b orthologues |
| --- | --- | --- | --- | --- |
| X | 1 | 18 | 25 | 28 |
| X | 4 | 23 | 22 | 18 |
| X | 8 | 16 | 8 | 9 |
| X | 18 | 23 | 14 | 6 |
| X | 18 | 24 | 26 | 4 |
| X | 23 | 24 | 27 | 4 |
| 1 | 1 | 3 | 14 | 7 |
| 1 | 1 | 4 | 9 | 7 |
| 1 | 1 | 10 | 17 | 9 |
| 1 | 1 | 18 | 49 | 14 |
| 1 | 4 | 5 | 7 | 21 |
| 1 | 4 | 6 | 17 | 9 |
| 1 | 5 | 14 | 72 | 8 |
| 1 | 6 | 9 | 14 | 20 |
| 1 | 8 | 12 | 115 | 126 |
| 2 | 1 | 4 | 2 | 8 |
| 2 | 2 | 4 | 53 | 66 |
| 2 | 2 | 9 | 2 | 6 |
| 2 | 2 | 16 | 4 | 4 |
| 2 | 4 | 16 | 40 | 16 |
| 2 | 4 | 17 | 13 | 8 |
| 2 | 6 | 9 | 50 | 50 |
| 2 | 10 | 20 | 14 | 16 |
| 2 | 19 | 20 | 30 | 5 |
| 3 | 1 | 3 | 7 | 12 |
| 3 | 1 | 4 | 2 | 8 |
| 3 | 1 | 11 | 29 | 45 |
| 3 | 1 | 19 | 5 | 10 |
| 3 | 3 | 11 | 3 | 16 |
| 3 | 4 | 16 | 11 | 10 |
| 3 | 5 | 15 | 16 | 14 |
| 3 | 8 | 13 | 2 | 6 |
| 3 | 12 | 15 | 2 | 4 |
| 4 | 2 | 9 | 3 | 11 |
| 4 | 6 | 17 | 8 | 25 |
| 4 | 7 | 10 | 13 | 23 |
| 4 | 8 | 10 | 16 | 58 |
| 5 | 5 | 14 | 11 | 10 |
| 5 | 6 | 17 | 65 | 113 |
| 5 | 18 | 24 | 85 | 25 |
| 6 | 5 | 14 | 7 | 13 |
| 6 | 5 | 15 | 6 | 2 |
| 6 | 6 | 9 | 32 | 107 |
| 6 | 7 | 15 | 2 | 8 |
| 6 | 9 | 19 | 72 | 23 |
| 6 | 19 | 20 | 10 | 8 |
| 7 | 1 | 24 | 2 | 4 |
| 7 | 2 | 3 | 31 | 21 |
| 7 | 2 | 16 | 6 | 6 |
| 7 | 3 | 7 | 13 | 2 |
| 7 | 4 | 17 | 4 | 4 |
| 7 | 5 | 14 | 17 | 24 |
| 7 | 7 | 15 | 2 | 9 |
| 7 | 18 | 24 | 11 | 2 |
| 8 | 4 | 17 | 39 | 16 |
| 8 | 5 | 14 | 55 | 33 |
| 8 | 6 | 9 | 4 | 9 |
| 8 | 12 | 15 | 45 | 7 |
| 8 | 21 | 23 | 2 | 4 |
| 9 | 1 | 11 | 5 | 7 |
| 9 | 4 | 17 | 3 | 9 |
| 9 | 6 | 17 | 59 | 80 |
| 9 | 7 | 10 | 15 | 6 |
| 10 | 2 | 16 | 2 | 10 |
| 10 | 8 | 16 | 5 | 3 |
| 10 | 8 | 19 | 2 | 7 |
| 10 | 16 | 19 | 44 | 29 |
| 10 | 16 | 20 | 25 | 26 |
| 10 | 19 | 20 | 17 | 5 |
| 11 | 2 | 18 | 13 | 29 |
| 11 | 7 | 10 | 12 | 3 |
| 11 | 7 | 25 | 35 | 43 |
| 11 | 8 | 13 | 4 | 12 |
| 11 | 13 | 24 | 52 | 45 |
| 11 | 18 | 24 | 13 | 9 |
| 12 | 2 | 3 | 53 | 56 |
| 12 | 3 | 7 | 15 | 4 |
| 12 | 4 | 23 | 3 | 22 |
| 12 | 6 | 17 | 19 | 21 |
| 12 | 18 | 21 | 14 | 23 |
| 13 | 1 | 4 | 11 | 12 |
| 13 | 4 | 16 | 48 | 2 |
| 14 | 6 | 9 | 218 | 18 |
| 14 | 21 | 23 | 2 | 14 |
| 15 | 6 | 9 | 50 | 2 |
| 15 | 7 | 15 | 6 | 6 |
| 15 | 7 | 25 | 12 | 46 |
| 15 | 15 | 25 | 50 | 79 |
| 16 | 15 | 25 | 83 | 46 |
| 16 | 19 | 22 | 50 | 64 |
| 17 | 1 | 19 | 2 | 8 |
| 17 | 8 | 19 | 9 | 23 |
| 17 | 13 | 24 | 24 | 41 |
| 17 | 19 | 22 | 16 | 9 |
| 17 | 19 | 23 | 18 | 5 |
| 17 | 23 | 24 | 6 | 4 |
| 18 | 6 | 17 | 6 | 31 |
| 18 | 12 | 15 | 9 | 6 |
| 18 | 15 | 21 | 32 | 8 |
| 19 | 1 | 3 | 2 | 4 |
| 19 | 1 | 12 | 11 | 87 |
| 19 | 5 | 13 | 61 | 34 |
| 19 | 8 | 19 | 21 | 11 |
| 19 | 15 | 25 | 4 | 8 |
| 19 | 19 | 23 | 6 | 3 |
| 20 | 3 | 11 | 2 | 11 |
| 20 | 6 | 9 | 6 | 6 |
| 20 | 10 | 20 | 10 | 15 |
| 20 | 11 | 16 | 25 | 8 |
| 21 | 1 | 4 | 7 | 12 |
| 21 | 1 | 24 | 7 | 4 |
| 22 | 2 | 3 | 5 | 5 |
| 22 | 6 | 17 | 16 | 24 |

**Supplementary Table 16. Oxford grid exhibition of the numbers of paralogues in golden arowana chromosomes.** Red cells imply paralogous chromosomes with more than 30 common paralogous genes.


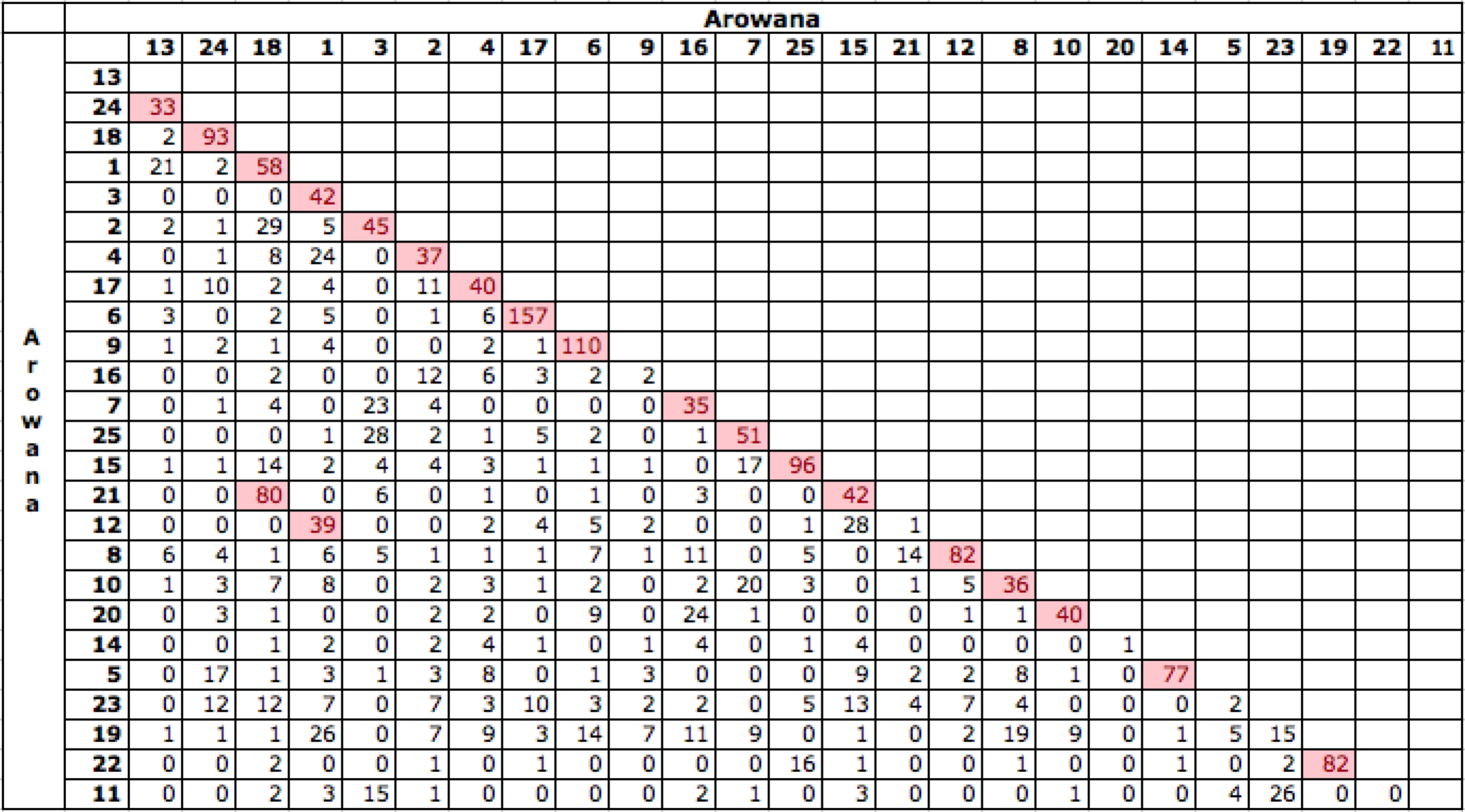


**Supplementary Table 17. Oxford grid showing the numbers of orthologues between golden arowana and medaka chromosomes.** Cells with more than 80 orthologues are highlighted in red.

**
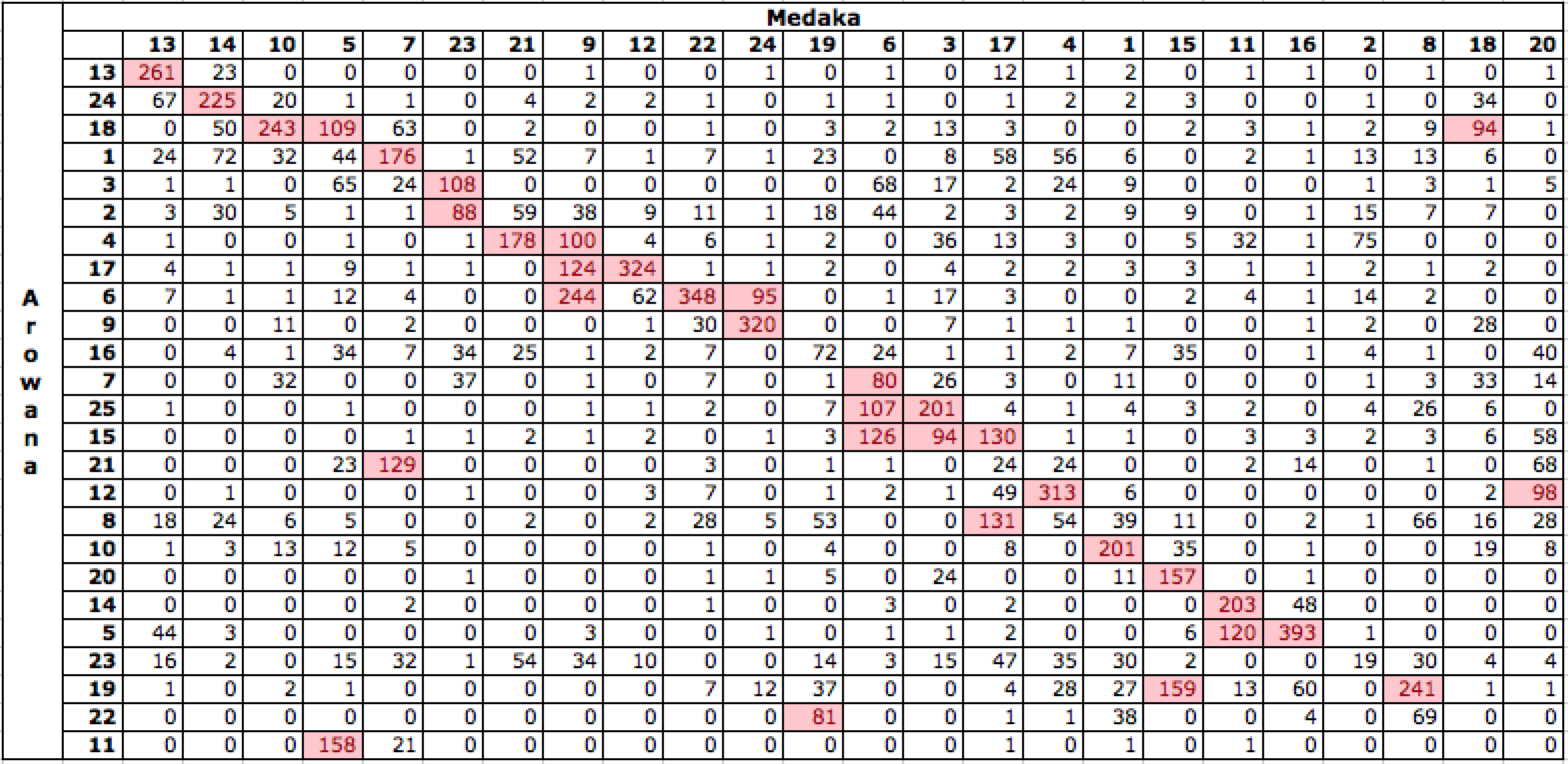
**

**Supplementary Table 18. Oxford grid showing the numbers of orthologues between gloden arowana and zebrafish chromosomes.** Cells with more than 80 orthologues are highlighted in red.

**
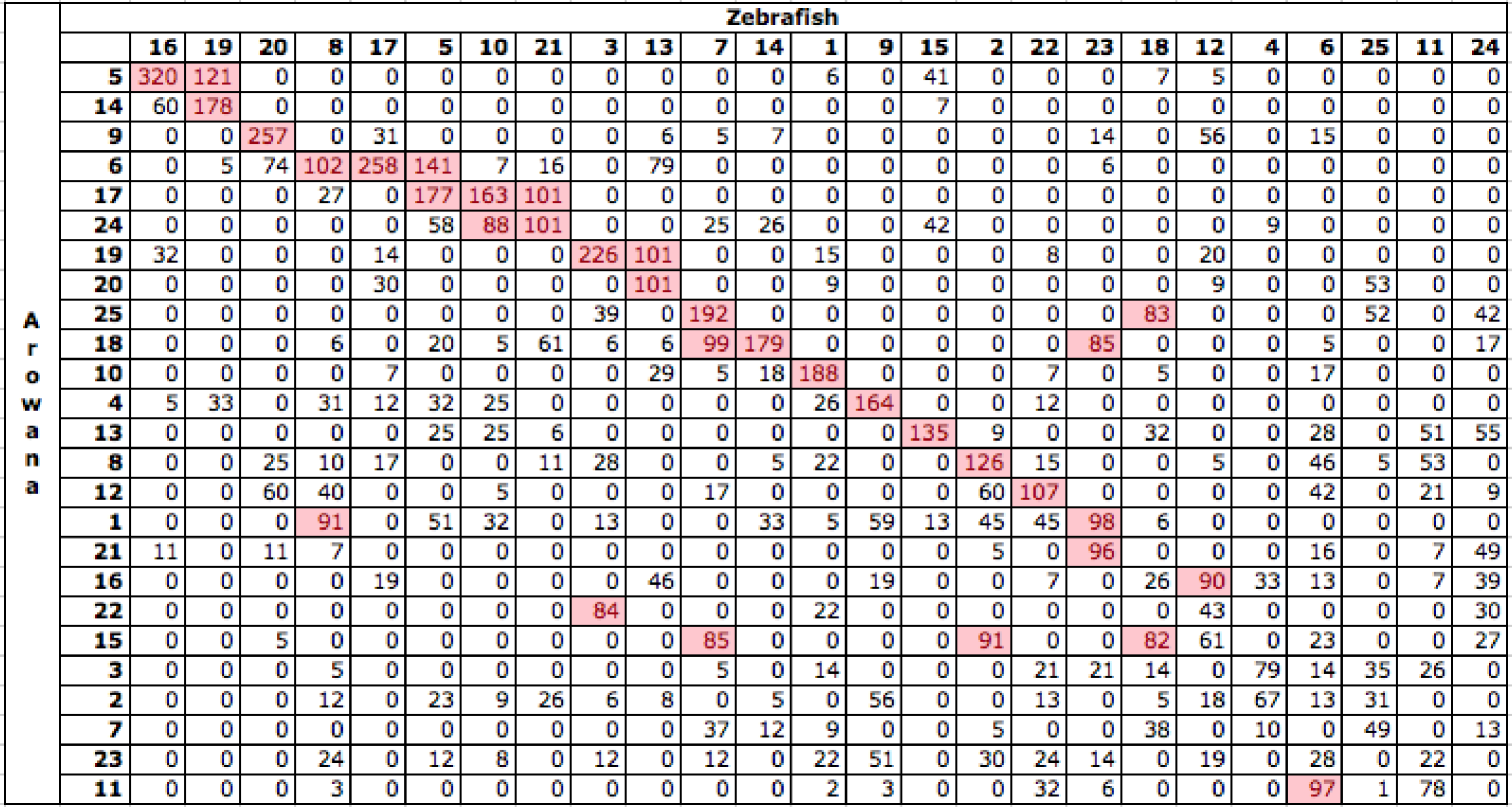
**

**Supplementary Table 19. Oxford grid showing the numbers of orthologues between golden arowana and spotted gar chromosomes.** Cells with more than 80 orthologues are highlighted in red.


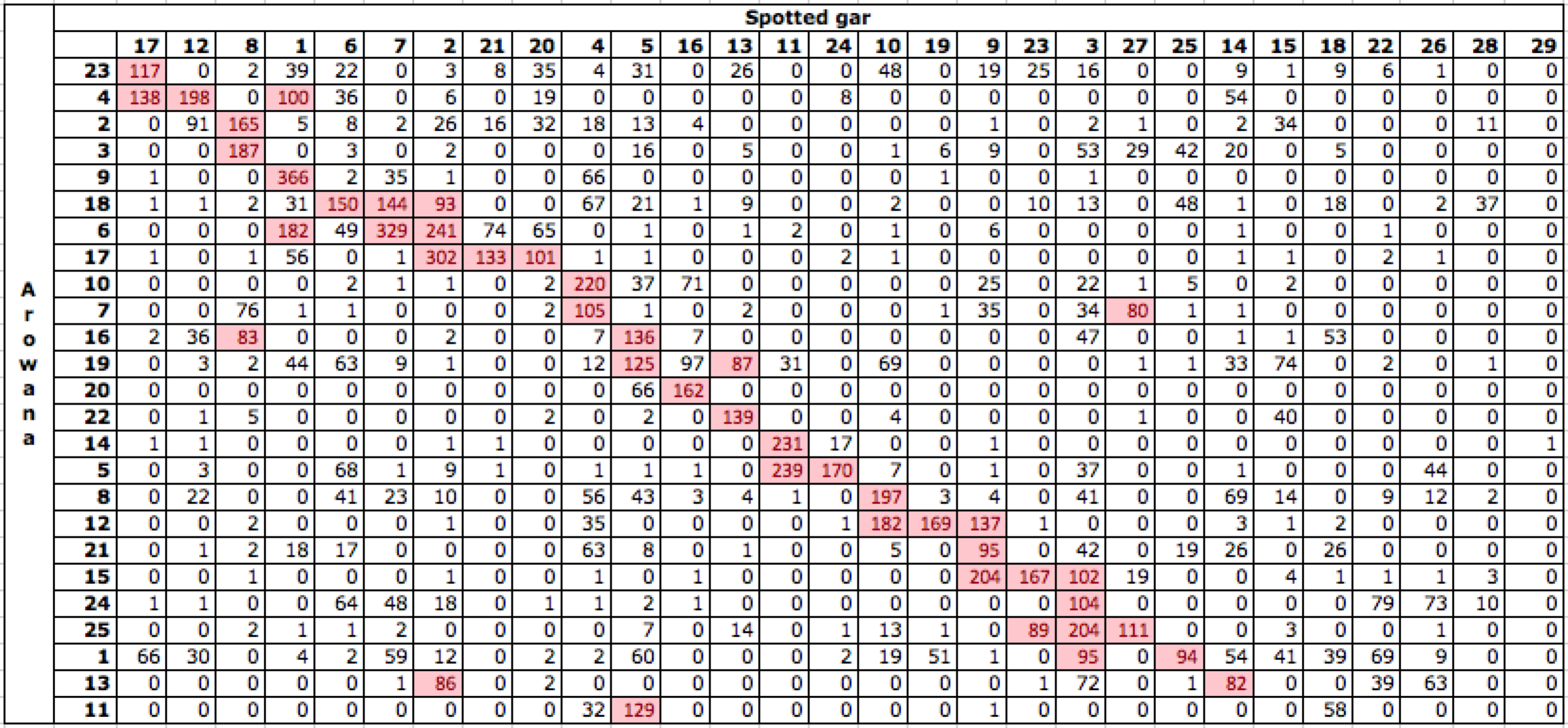


**Supplementary Table 20. Major interchromosomal rearrangements in arowana, zebrafish and medaka genomes in relation to the linkage groups (LGs) of spotted gar.** Respective Circos plots are shown in Figure 3B,C and D. As a result of the teleost-specific whole-genome duplication event, one would expect a 1:2 relation between spotted gar chromosomes and teleost chromosomes. Numbers higher than that indicate rearrangement events in the teleost fishes.

| **Spotted Gar LG** | **Number of interchromosomal rearrangements** | | |
| --- | --- | --- | --- |
|  | **Arowana** | **Zebrafish** | **Medaka** |
| 1 | 9 | 9 | 8 |
| 2 | 7 | 8 | 6 |
| 3 | 11 | 14 | 7 |
| 4 | 9 | 11 | 7 |
| 5 | 9 | 10 | 6 |
| 6 | 7 | 10 | 7 |
| 7 | 6 | 8 | 6 |
| 8 | 4 | 5 | 3 |
| 9 | 5 | 6 | 5 |
| 10 | 6 | 10 | 6 |
| 11 | 3 | 4 | 2 |
| 12 | 4 | 7 | 6 |
| 13 | 6 | 6 | 3 |
| 14 | 6 | 9 | 7 |
| 15 | 5 | 4 | 2 |
| 16 | 3 | 6 | 4 |
| 17 | 3 | 7 | 6 |
| 18 | 6 | 4 | 2 |
| 19 | 2 | 4 | 3 |
| 20 | 4 | 4 | 2 |
| 21 | 4 | 5 | 2 |
| 22 | 2 | 4 | 2 |
| 23 | 2 | 4 | 2 |
| 24 | 2 | 3 | 2 |
| 25 | 4 | 5 | 2 |
| 26 | 2 | 6 | 4 |
| 27 | 1 | 3 | 2 |
| 28 | 1 | 3 | 2 |
| 29 | 0 | 0 | 0 |
| Total | 133 | 179 | 116 |

**Supplementary Table 21. Asian arowana samples used for karyotype and RNAseq analysis**

| N | Tag number | ID | Sex |
| --- | --- | --- | --- |
| 1 | 134547673A | ACN03 | Male |
| 2 | 135433114A | ACY07 | Male |
| 3 | 142214564A | AEJ03 | Male |
| 4 | 0A00382515 | AGB10 | Male |
| 5 | 134534333A | AGC01 | Male |
| 6 | 134676112A | AGD08 | Male |
| 7 | 145223194A | AGD10 | Male |
| 8 | 146659117A | AGE02 | Male |
| 9 | 127279231A | AGE10 | Male |
| 10 | 0A00186337 | AIC09 | Male |
| 11 | 145223111A | ACT03 | Female |
| 12 | 144751331A | AEG02 | Female |
| 13 | 702088880029940 | AES07 | Female |
| 14 | 0A00406414 | AGB04 | Female |
| 15 | 0A00620355 | AGD01 | Female |
| 16 | 145146330A | AGD06 | Female |
| 17 | 134647372A | AGE09 | Female |
| 18 | 0A00383253 | AGF02 | Female |
| 19 | 147239196A | AHO02 | Female |
| 20 | 5555013300 | AIQ10 | Female |
| 21 | [1828197826](callto:1828197826) |  | Male |
| 22 | 146552361A |  | Male |
| 23 | 702018180023030 |  | Male |
| 24 | [6818205374](callto:6818205374) |  | Female |
| 25 | [1828197338](callto:1828197338) |  | Female |
| 26 | 127626265A |  | Female |

* Records 1-20 are the fin clips of Asian arowana Tong Yan hybrids used for karyotype analysis; 21-25 are the six adult Asian arowanas of the golden variety from Qian Hu Fish Farm used for RNASeq and qPCR validation.

**Supplementary Table 22. Primers used for rDNA analysis and the qPCR validation of the RNAseq results**

| **Gene ID** | **Gene name** | **Chr** | **Forward primer (5’-3’)** | **Reverse primer (5’-3’)** |
| --- | --- | --- | --- | --- |
| / | 18S rDNA | / | GCGAAGGGTAGACACACGCTGA | CCTCTAGCGGCACAATACGAATG |
| / | 5S rDNA | / | TACGCCCGATCTCGTCCGATC | CGGCTGGTATGGCCGTAAGC |
| / | gal3st | / | GTCATCCAGATCCCAGGAGA | GCCAGATGAAAAGGACTCCA |
| AR_GLEAN_10000825 | *tbx3* | 2 | CGAAGTCTGGAAGGCGAATG | CCACGATGTCCATCAGCAAG |
| AR_GLEAN_10006813 | *cers6* | 2 | ACTTGTCCCTGCTCTTCTCC | CATGAAGGCACATGACGAGG |
| AR_GLEAN_10004113 | *bgal* | 4 | TGGCAGCTATTTTGCCTGTG | TCCCATCGGTGGTAAAGAGG |
| AR_GLEAN_10005912 | *kgp1* | 4 | ATGTCACCCTGACCAGCTAC | GCCAAGATCTGCCCAACTTC |
| AR_GLEAN_10006238 | *cyt* | 4 | AAGAGAGCTGATGTCGGTGG | TGATCGGTTGAGGAGCCTTT |
| AR_GLEAN_10007202 | *trm2a* | 4 | AAAAGCCAGGAGGAAAGGGA | TTGAGGATGGGGTCAGCTTT |
| AR_GLEAN_10007243 | *ckp2l* | 4 | AACTGTCCCCTCTAAAGCCC | ACCGGTGGATTGACCTTCAT |
| AR_GLEAN_10005771 | *fhl3* | *5* | TGTGGCCTGTGACAAAACTG | ACGCAGTAGTGCTCATCCTT |
| AR_GLEAN_10003181 | *hsc20* | 17 | TATATGCTGGAGCTGGTGGG | GCTTTGGCTTCCTCTTTGCT |
| AR_GLEAN_10006600 | *chss3* | 17 | ACCGTGCTTCAGGTAATGGA | GCGGTAGCCATACTGGATCT |
| AR_GLEAN_10009027 | *gcnfa* | 17 | GCATGAACCGCAAAGCAATC | ACACCCTCCTTGAACTCCTG |
| AR_GLEAN_10021185 | *swahb* | 17 | CGGGTTCTCAACACGCTATG | ACCAGAAGGCGGATCATCTT |
| AR_GLEAN_10011097 | *aapk1* | 17 | CTAGGGGTAGGGACGTTTGG | CAGGCTGCGAATCTTCTGTC |
| AR_GLEAN_10011185 | *dmrt3a* | *17* | TCCTCCAGCACACTTACCAG | AAAATGGGTTGGCCTGGTTC |
| AR_GLEAN_10019799 | *sf01* | *24* | AAAGTTGGAGGAGGAGAGGC | TTGTAATCGGAAGGGGGCTT |

**Supplementary Table 23. The distribution of reads generated from fragments microdissected from the five chromosomes when mapped to the golden Asian arowana chromosomes**

| **Gloden arowana**  **chromosomes** | **AroW1 & AroW3** | | **AroA4** | | **AroA1** | | **AroA2** | |
| --- | --- | --- | --- | --- | --- | --- | --- | --- |
|  | **Mapped**  **reads** | **%** | **Mapped**  **reads** | **%** | **Mapped**  **reads** | **%** | **Mapped**  **reads** | **%** |
| **Chr1** | 5,864 | 2.5 | 17,951 | 20.6 | 863 | 2.3 | 597 | 1.4 |
| **Chr2** | 52,433 | 22.5 | 1,238 | 1.4 | 636 | 1.7 | 474 | 1.1 |
| **Chr3** | 3,253 | 1.4 | 26,879 | 30.8 | 425 | 1.1 | 393 | 0.9 |
| **Chr4** | 107,821 | 46.3 | 1,524 | 1.7 | 853 | 2.3 | 601 | 1.4 |
| **Chr5** | 1,836 | 0.8 | 1,201 | 1.4 | 511 | 1.4 | 378 | 0.9 |
| **Chr6** | 4,099 | 1.8 | 2,077 | 2.4 | 1,032 | 2.8 | 764 | 1.8 |
| **Chr7** | 2,783 | 1.2 | 550 | 0.6 | 271 | 0.7 | 167 | 0.4 |
| **Chr8** | 1,637 | 0.7 | 1,192 | 1.4 | 448 | 1.2 | 430 | 1.0 |
| **Chr9** | 1,303 | 0.6 | 1,266 | 1.5 | 12,324 | 33.3 | 404 | 0.9 |
| **Chr10** | 2,723 | 1.2 | 819 | 0.9 | 437 | 1.2 | 430 | 1.0 |
| **Chr11** | 741 | 0.3 | 326 | 0.4 | 163 | 0.4 | 140 | 0.3 |
| **Chr12** | 1,748 | 0.8 | 4,506 | 5.2 | 477 | 1.3 | 34,187 | 78.6 |
| **Chr13** | 5,748 | 2.5 | 1,054 | 1.2 | 475 | 1.3 | 269 | 0.6 |
| **Chr14** | 764 | 0.3 | 688 | 0.8 | 178 | 0.5 | 132 | 0.3 |
| **Chr15** | 2,855 | 1.2 | 2,525 | 2.9 | 659 | 1.8 | 506 | 1.2 |
| **Chr16** | 2,062 | 0.9 | 769 | 0.9 | 321 | 0.9 | 320 | 0.7 |
| **Chr17** | 15,339 | 6.6 | 1,276 | 1.5 | 780 | 2.1 | 302 | 0.7 |
| **Chr18** | 2,643 | 1.1 | 1,021 | 1.2 | 477 | 1.3 | 543 | 1.2 |
| **Chr19** | 2,697 | 1.2 | 1,013 | 1.2 | 620 | 1.7 | 340 | 0.8 |
| **Chr20** | 1,784 | 0.8 | 2,923 | 3.4 | 167 | 0.5 | 190 | 0.4 |
| **Chr21** | 1,269 | 0.5 | 900 | 1.0 | 428 | 1.2 | 316 | 0.7 |
| **Chr22** | 4,041 | 1.7 | 11,096 | 12.7 | 159 | 0.4 | 124 | 0.3 |
| **Chr23** | 2,714 | 1.2 | 1,556 | 1.8 | 13,320 | 36.0 | 756 | 1.7 |
| **Chr24** | 1,882 | 0.8 | 1,714 | 2.0 | 573 | 1.6 | 394 | 0.9 |
| **Chr25** | 2,887 | 1.2 | 1,132 | 1.3 | 363 | 1.0 | 335 | 0.8 |
| **Total** | 232,926 | / | 87,196 | / | 36,960 | / | 43,492 | / |

**Supplementary Table 24. Assembly statistics of the sequences generated from fragments microdissected from the five chromosomes (30kb chains).**

| **Name of microdissected chromosomes** | **Number of pseudo-scaffolds** | **Length (bp)** | **Repeat in the assembly (%)** | **Number of predicted genes** | **Gene density (/Mb)** |
| --- | --- | --- | --- | --- | --- |
| **AroW1** | 1,016 | 37,289,956 | 0.765 | 427 | 11.45 |
| **AroW3** | 801 | 8,373,719 | 1.479 | 341 | 40.74 |
| **AroA1** | 972 | 986,651 | 1.098 | N/D | N/D |
| **AroA2** | 559 | 14,823,933 | 0.893 | 220 | 14.84 |
| **AroA4** | 1,144 | 10,354,760 | 1.282 | 252 | 24.34 |

**Supplementary Table 25. Genes upregulated in testis and ovary, their position on the chromosomes as well as their related function**

**(see separate Excel file)**

1. Benton MJ, Donoghue PC: **Paleontological evidence to date the tree of life.** *Mol Biol Evol* 2007, **24:**26-53.

2. Hurley IA, Mueller RL, Dunn KA, Schmidt EJ, Friedman M, Ho RK, Prince VE, Yang Z, Thomas MG, Coates MI: **A new time-scale for ray-finned fish evolution.** *Proc Biol Sci* 2007, **274:**489-498.

3. Kumazawa Y, Nishida M: **Molecular phylogeny of osteoglossoids: a new model for Gondwanian origin and plate tectonic transportation of the Asian arowana.** *Mol Biol Evol* 2000, **17:**1869-1878.

4. Botella H, Blom H, Dorka M, Ahlberg PE, Janvier P: **Jaws and teeth of the earliest bony fishes.** *Nature* 2007, **448:**583-586.

5. Robinson MD, McCarthy DJ, Smyth GK: **edgeR: a Bioconductor package for differential expression analysis of digital gene expression data.** *Bioinformatics* 2010, **26:**139-140.
